# The impact of non-neutral synonymous mutations when inferring selection on nonsynonymous mutations

**DOI:** 10.1093/genetics/iyaf200

**Published:** 2025-09-27

**Authors:** Aina Martinez i Zurita, Christopher C Kyriazis, Kirk E Lohmueller

**Affiliations:** Department of Human Genetics, David Geffen School of Medicine, University of California, Los Angeles, CA 90095-1606, United States; Department of Ecology and Evolutionary Biology, University of California, Los Angeles, CA 90095-1606, United States; Department of Human Genetics, David Geffen School of Medicine, University of California, Los Angeles, CA 90095-1606, United States; Department of Ecology and Evolutionary Biology, University of California, Los Angeles, CA 90095-1606, United States; Interdepartmental Program in Bioinformatics, University of California, Los Angeles, CA 90095-1606, United States

**Keywords:** DFE, demographic inference, synonymous mutations, population genetics

## Abstract

The distribution of fitness effects (DFE) describes the proportions of new mutations that have different effects on fitness. Accurate measurements of the DFE are important because the DFE is a fundamental parameter in evolutionary genetics and has implications for understanding of other phenomena such as complex disease or inbreeding depression. Current computational methods to infer the DFE for nonsynonymous mutations from natural variation first estimate demographic parameters from synonymous variants to control for the effects of demography and background selection. Then, conditional on these parameters, the DFE is inferred for nonsynonymous mutations. This approach relies on the assumption that synonymous variants are neutrally evolving. However, some evidence points toward synonymous mutations having measurable effects on fitness. To test whether selection on synonymous mutations affects inference of the DFE of nonsynonymous mutations, we simulated several possible models of selection on synonymous mutations using SLiM and attempted to recover the DFE of nonsynonymous mutations using Fit∂a∂i, a common method for DFE inference. Our results show that the presence of selection on synonymous variants leads to incorrect inferences of recent population growth. Furthermore, under certain parameter combinations with pervasive selection on synonymous mutations, the inferred DFEs for nonsynonymous mutations show an inflated proportion of highly deleterious and nearly neutral mutations. However, this bias can be eliminated if the correct demographic parameters are used for DFE inference instead of the biased ones inferred from synonymous variants. Our work demonstrates how unmodeled selection on synonymous mutations may affect downstream inferences of the DFE.

## Introduction

Mutations can have different effects on the fitness of an individual, ranging from a fitness benefit to being neutral with respect to the chances of survival of the individual, or causing harmful or even lethal effects on the carrier. The distribution of fitness effects (DFE) is a probability distribution that describes the relative abundances of new mutations in each category (Eyre-Walker and Keightley 2007). That is, the DFE describes the expected abundance of mutations with a particular fitness effect.

Understanding the DFE of new mutations is fundamental for both practical and theoretical questions. From a basic biology standpoint, the DFE directly describes how selection might affect the maintenance of genetic variation in a population. In addition, the DFE of a given species impacts our understanding of the molecular clock (Ohta 1992) and has implications for the evolution of sex and recombination (Keightley and Otto 2006). From a practical standpoint, having a reasonable estimate of the DFE for new mutations helps in understanding the amount of phenotypic variance a given mutation could explain, in turn affecting the genetic architecture of complex disease (Eyre-Walker et al. 2006; Eyre-Walker 2010). In addition, DFE estimates can inform our decision-making in the management of small populations of plants or animals in conservation efforts (Kyriazis et al. 2020, 2023; Robinson et al. 2023). In summary, accurate DFE estimates for new mutations impact many fundamental aspects of modern population genetics.

There have been two main approaches used to estimate the DFE of new mutations. The first type uses direct experimental measurements applied to microorganisms, conducted either via mutagenesis experiments or mutation accumulation experiments (Elena et al. 1998; Bataillon and Bailey 2014). Experimental approaches allow for direct measurement of the DFE. However, they are limited to organisms that are amenable to experimental manipulation, which severely limits the range of species for which the DFE can be estimated. Given the existing evidence of differences in the DFE between species (Huber et al. 2017), estimates from microorganisms are insufficient to properly characterize the DFE of higher-order species.

A second class of methods applies computational evolutionary models to polymorphism data derived from natural populations (Eyre-Walker et al. 2006; Keightley and Eyre-Walker 2007; Boyko et al 2008; Kim et al. 2017; Tataru and Bataillon 2019). This suite of methods estimates the DFE that predicts the polymorphism data summarized by the site-frequency spectrum (SFS), or numbers of variants at different allele frequencies in the sample of individuals (Eyre-Walker et al. 2006; Keightley and Eyre-Walker 2007; Kim et al. 2017; Tataru and Bataillon 2019 ). Using these methods, researchers have estimated the DFE of nonsynonymous mutations in humans (Boyko et al. 2008; Li et al. 2010; Kim et al. 2017; Huang et al. 2021) as well as numerous other taxa including animals and plants (Galtier 2016; Chen et al. 2017; Castellano et al. 2019).

The SFS of nonsynonymous variants is shaped by selective pressures as well as demographic processes (Williamson et al. 2005). Hence, methods need to control for demographic history in order to infer the DFE parameters. The majority of methods follow a similar workflow (Supplementary Fig. 1). First, the genetic variation data is divided in two classes of sites: one putatively neutral and the other potentially experiencing the effects of selection (eg nonsynonymous sites). Both categories are summarized into their respective SFS. Then, the neutral SFS is used to infer the effect of demographic processes that could influence the SFS. Finally, conditioning on the demographic history, a DFE is fit to the SFS from the variants potentially experiencing selection. The deviation in the SFS of the variants putatively under selection from the SFS expected under the demographic model is used to fit the parameters of the DFE.

Historically, synonymous variants have been assumed to be effectively neutral since they lack an effect on the amino acid sequence (Williamson et al. 2005; Bailey et al. 2021). Following this logic, it is standard to use synonymous variants as a neutral reference to estimate demographic history prior to performing DFE inference (Boyko et al. 2008; Galtier 2016; Kim et al. 2017). The use of synonymous variants also helps account for background selection between nonsynonymous variants that could be skewing the nonsynonymous SFS, since synonymous variants are interdigitated in the genome and therefore under the same background selection pressure (Kim et al. 2017).

However, several lines of evidence point toward synonymous mutations potentially experiencing selection (reviewed in Zhang and Qian 2025). Experimental studies conducted in a range of microorganisms have found evidence of synonymous mutations causing both deleterious and beneficial selective effects (Bailey et al. 2021). Widespread evidence of codon bias across different taxa suggests synonymous mutations being impacted by selective forces (Carlini and Stephan 2003; Hershberg and Petrov 2008). Some synonymous mutations have been identified as having significant contributions to human disease (Sauna and Kimchi-Sarfaty 2011); however, the overall impact of synonymous mutations on human disease may be minor (Dhindsa et al. 2022). Studies from both human and *Drosophila* populations have found evidence of a percentage of synonymous mutations being under strong purifying selection (Keightley and Halligan 2011; Lawrie et al. 2013; Ragsdale et al. 2018; Machado et al. 2020). In humans, several studies have inferred a weakly deleterious DFE for synonymous mutations using intragenic regions or codon-flanking sites as a putatively neutral control (Ragsdale et al. 2018; Huang and Siepel 2019). Finally, some recent experimental results show widespread selection acting on synonymous mutations (Shen et al. 2022a); however, the validity of these results is under debate (Shen et al. 2022b; Kruglyak et al. 2023). Nevertheless, the effect of selection on synonymous mutations on DFE inference of nonsynonymous mutations remains unclear.

In this work, we address this knowledge gap by testing the robustness of one DFE inference method, Fit∂a∂i (Kim et al. 2017), to the presence of selection on synonymous mutations. First, we show that selection on synonymous mutations results in false evidence of population expansion events during the demographic inference step. Furthermore, we show that the selection acting on synonymous mutations can sometimes affect the DFE parameter estimates for nonsynonymous mutations. Finally, we show that the misestimation of the demographic parameters is primarily responsible for the biased DFE inferences. This points toward a possible strategy to resolve the bias as well as a potential approach for detecting selection acting on synonymous mutations.

## Methods

### Simulations with constant population size

To test the performance of Fit∂a∂i in the presence of selection on synonymous mutations, we simulated datasets with known selection parameters. Simulations were performed with the forward-in-time evolutionary simulation framework, SLiM 3 (Haller and Messer 2019). A population with a constant size of 10,000 individuals was simulated for 100,000 burn-in generations, followed by an additional 1,000 generations before sampling 100 haploid genomes. For each simulation run, random exon and intron regions were generated across the total length of the simulated sequence with an average 1.4 Mb in exonic sequence and 19.6 Mb in intronic sequence. The mutation rate (*μ*) and recombination rate (*r*) were constant across the entire length of the sequence with *μ* = 1.5e-8 per base pair per generation and *r* = 1e-8 per base position per generation, unless otherwise specified. Further simulation parameters are listed in Supplementary Table 1.

Two categories of mutations were simulated in the exonic regions: nonsynonymous (NS) mutations and synonymous (S) mutations. The ratio of NS to S mutations in the simulation was 2.31:1 (Huber et al. 2017). We simulated a human nonsynonymous DFE (Kim et al. 2017) where the deleterious selection coefficients (*s*) for NS mutations were drawn from a gamma distribution with a scale parameter (in terms of 2*Ns*) of −706.899 and a shape parameter of 0.186, yielding a mean *s* of −0.013. The selection coefficients for the synonymous mutations depended on the model being tested. Under the simplest model, the constant model, all synonymous mutations were assigned the same deleterious selection coefficient. Three values of *s* were tested: *s* = 1e-5 (*Ns* < 1, nearly neutral), *s* = 1e-4 (*Ns* ∼ 1, weakly deleterious), and *s* = 1e-3 (*Ns* ~ 10, strongly deleterious). Under the partial model, 22% of synonymous mutations were assigned the same selection coefficient, of *s* = 1e-5, *s* = 1e-4, or *s* = 1e-3. The remaining 78% of mutations were neutrally evolving and had *s* = 0. The partial model reflects an attempt at a more biologically informed model for selection on synonymous mutations based on estimates from *Drosophila melanogaster* (Lawrie et al. 2013). Accounting for the different synonymous selection coefficients, we conducted simulations for a total of six conditions with selection on synonymous mutations. We also simulated a control condition where all synonymous mutations were neutral (*s* = 0).

Each individual simulation run contained approximately 1.5 Mb of coding sequence. To generate the data for a single replicate, we aggregated the results of 22 individual simulations, run in parallel to speed up computation. The results of each individual run were aggregated into a synonymous and nonsynonymous SFS. The respective SFS of 22 simulations were summed to obtain a single replicate synonymous and nonsynonymous SFS. For each condition, we generated 20 replicates, where each replicate represents approximately 30 Mb of coding sequence (1.5 Mb per simulation × 22 parallel simulations ∼ 30 Mb).

### Simulations with increased recombination rate

To test the effects of linkage on our results, we increased the rate of recombination. We considered 2 scenarios with a ∼10× increase in recombination rate with respect to the mutation rate (*r* = 1e-7) and a ∼100× increase (*r* = 1e-6). We ran the simulations as described in the previous section, varying only the recombination parameter. We generated 20 replicates with each of the 2 elevated recombination rates, totaling 14 distinct possible simulated parameter combinations (selection scenario × recombination rate).

### Human-like simulations

To explore more realistic scenarios using parameters derived from humans, we simulated a human-like demographic history with four populations modeling the out-of-Africa expansion described by Gravel et al. (2011). We used recent estimates for the population sizes, epoch times, and migration rates reported by Jouganous et al. (2017) (Supplementary Table 2; Methods). The simulations started with a burn-in period of 112,930 generations, followed by 12,312 additional generations before sampling 100 haploid genomes. For each individual simulation, we approximated a human-like chromosomal region by randomly sampling exon lengths and noncoding region lengths from chromosome 1 in the GRCh38.p14 human genome assembly annotation in GENCODE Release 45. The *μ* and *r* were constant across the entire length of the sequence with *μ* = 1.44e-8 (Gravel et al. 2013) per base pair per generation and *r* = 1e-8 per base position per generation. Further simulation parameters are listed in Supplementary Table 2.

We used the same NS:S ratio and DFE for nonsynonymous mutations as described previously. For the synonymous mutations, we simulated two possible scenarios: one where all synonymous mutations are neutral, *s* = 0, and one where values of *s* for synonymous mutations are deleterious and drawn from a gamma distributed DFE. We employed an estimate of the DFE for synonymous mutations in humans described in Ragsdale et al. (2018), derived from a sample of 797 French–Canadian individuals. Ragsdale et al. (2018) estimated a scale parameter (in terms of 2*Ns*) of −55 and shape parameter of 0.14; yielding a mean *s* of −0.00068.

For each possible scenario of selection on synonymous mutations, we generated 10 replicate simulations. Each simulation replicate contained 800 Mb of simulated sequence, which was generated by aggregating 200 individual simulated fragments of 4 Mb run in parallel to speed up computation. For each simulation replicate, the nonsynonymous and synonymous SFSs of the African and European populations were tallied up at the end of the simulation and used for subsequent Fit∂a∂i analysis.

### Quantifying the magnitude of background selection

To investigate the magnitude of the effects of background selection on our simulations, we used the ratio of the expected average number of pairwise differences under neutral evolution (π_0_) vs the measured amount from our simulated data (π). For a given set of simulated parameters and mutational class (synonymous or nonsynonymous), we computed π_0_ as 4*N*μ*L*, where μ is the per-base-pair mutation rate, *L* is the length of possible synonymous (*L*_s_) or nonsynonymous (*L*_ns_) sequence, and *N* is the size of the population. We calculated π_0_ to be 6e-4 per site. When multiplied by the length of the nonsynonymous and synonymous sequence, we obtained an average of 13,479.52 and 5836.84 expected total pairwise differences, respectively.

We then computed π for each simulation replicate. π was estimated from the SFS using the built-in ∂a∂i package function Spectrum.pi. The ratio of π/π_0_ was obtained by dividing the obtained in each simulation replicate by the corresponding synonymous or nonsynonymous π_0_.

### Demographic inference

We used the software package ∂a∂i (Gutenkunst et al. 2009) to infer the demographic parameters that provided the best fit to the synonymous SFS in both the constant population and human-like simulations. We fit two possible demographic models to the data: a 1-epoch model, which corresponds to a constant population size throughout the entirety of the population's history, and a 2-epoch model, where a single population size change occurred at some point in the population's history.

We used a likelihood ratio test (LRT) to determine whether the 2-epoch model fit the data significantly better than the 1-epoch model. The log-likelihood ratio test statistic between the null (1-epoch) and alternative (2-epoch) models can be asymptotically approximated under the null hypothesis by a χ^2^ distribution with 2 degrees of freedom (Wilks 1938). A log-likelihood difference of >3 represents a significance level of 0.05 and allowed us to reject the 1-epoch model in favor of the 2-epoch model for providing a statistically significant better fit to the data (Adams and Hudson 2004).

For each simulation replicate, we first tested 25 possible initial parameters in each round of model fitting. Initial parameters were tweaked until a good fit between the simulation SFS, and the predicted SFS under the demographic model was achieved. Satisfactory fit was assessed through visual inspection of the observed and predicted SFS for all demographic inferences for all replicates. Plots showing satisfactory fits of the true and predicted SFS for each simulation replicate are available on the GitHub repository associated with this work. We imposed a range of possible values for the 2-epoch demographic parameters. Specifically, we allowed *v* (fold difference between current and ancestral population sizes) to range from 0 to 3000, allowing for the possibility of a demographic contraction (*v* < 1) or expansion (*v* > 1). An upper bound of 3,000 was chosen because it corresponds to small values of the population-scaled synonymous mutation rate, θ_s_ ∼ 1.5, which in turn represents a highly biologically improbable scenario of an ancestral population size of ∼3 individuals. We allowed *T* (time of fold change in units of *2N_a_* generations) to range from 0 to 500.

### DFE inference

We used the software package Fit∂a∂i (Kim et al. 2017) to infer the parameters of the DFE of the nonsynonymous mutations in both the constant population and human-like simulations. We conditioned the inference on the best-fit demographic parameters obtained for each simulation replicate after following the inference procedure outlined above. We fit a gamma-distributed DFE to the nonsynonymous SFS, which was the functional form of the true DFE used to generate the data. For each replicate, we conducted 25 independent iterations of model fitting, varying the initial gamma distribution parameters. The parameter values having the maximum likelihood of all the iterations were considered the best fit.

**Fig. 1.**
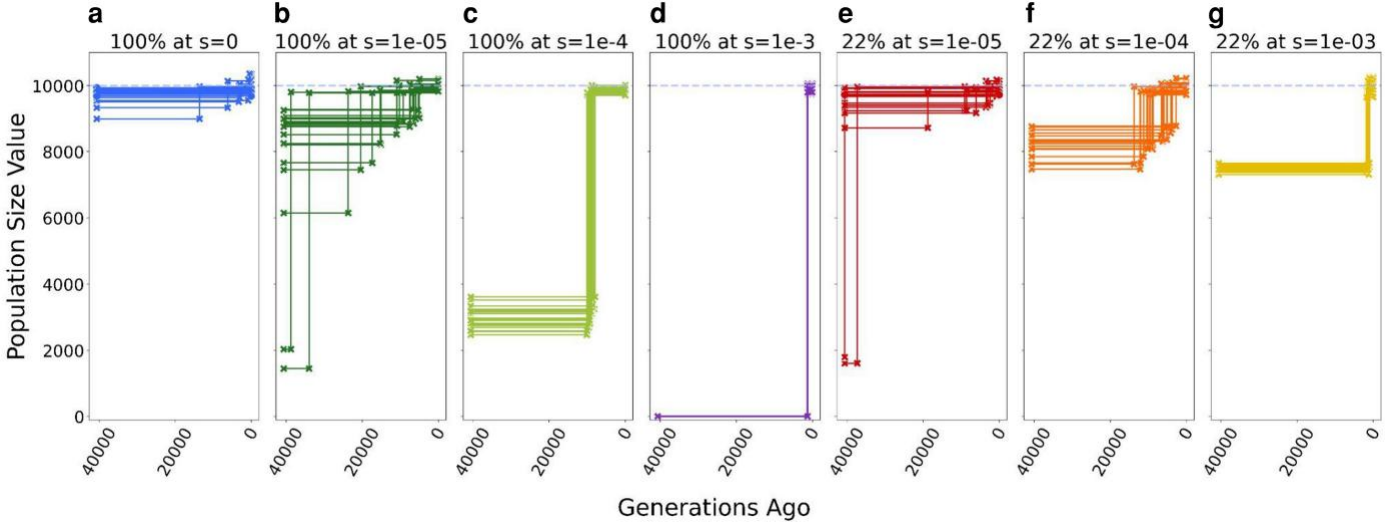
Inference of demography with varying degrees of selection on synonymous mutations. Inferred population size for each replicate under each model of selection on synonymous mutations. Each scenario includes 20 simulation replicates. When a 2-epoch model (1 population size change at a specific time in the past) provided the best fit, the inferred time of the demographic event is indicated by a step in the plot between the ancestral population size and current population size. A horizontal line indicates data best described by a one-epoch (constant population size) model. Dashed line corresponds to the true population size in all simulations (*N* = 10,000). Inference in each replicate was performed on a sample of 100 chromosomes. a) Neutral model, with no selection on synonymous mutations (*s* = 0). b to d) Models where all synonymous mutations experienced selection, with deleterious selection coefficients (*s*) ranging from 1e-5 to 1e-3. e to g) Model where 22% of synonymous mutations experienced selection, with deleterious selection coefficients (*s*) ranging from 1e-5 to 1e-3. The remaining 78% of synonymous mutations were neutral (*s* = 0).

**Fig. 2.**
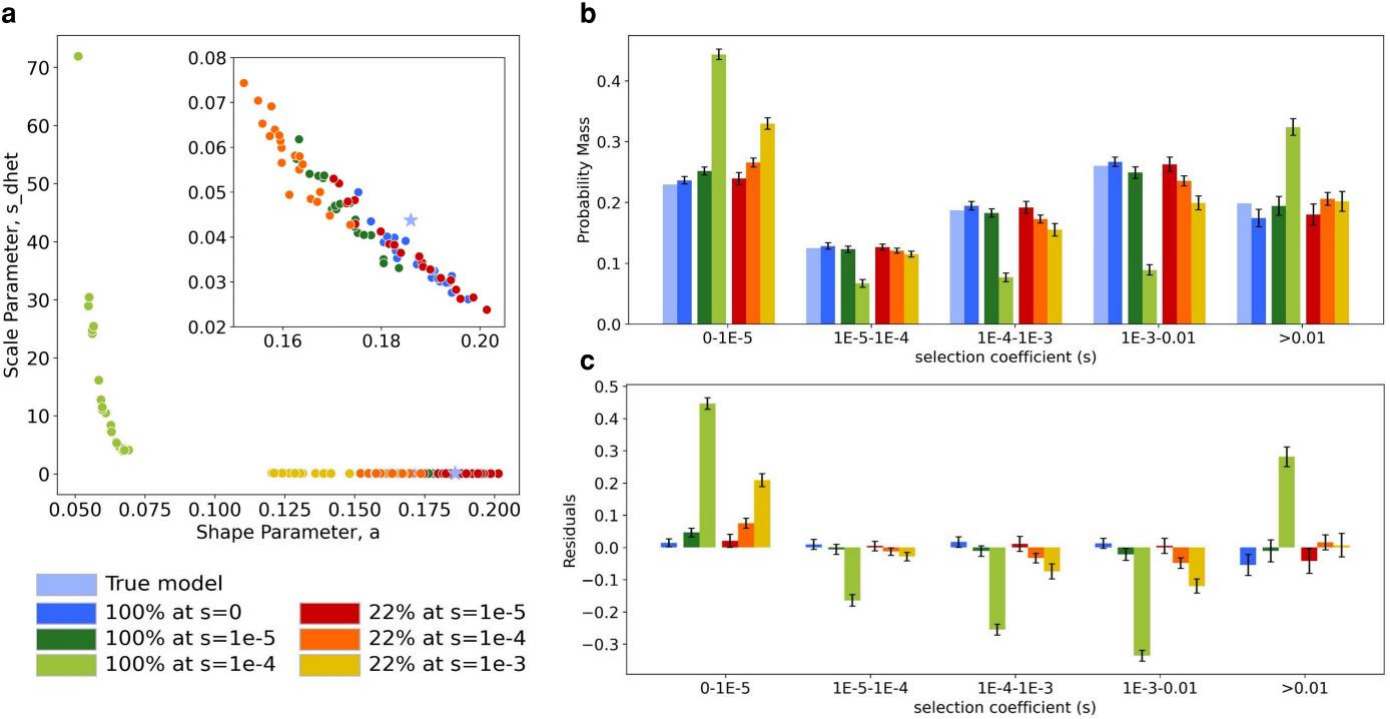
Inference of the DFE for nonsynonymous mutations under different models of selection on synonymous mutations. a) Inferred shape and scale parameters of a gamma DFE model for nonsynonymous mutations from simulated data with distinct levels of selection on synonymous mutations. Each point represents an individual simulation replicate. Scale parameter, *s_dhet_*, represents the scale parameter in units of heterozygous selection strength. Insert zooms in on the lower-right section of the plot. b) Comparison of the discretized DFE for nonsynonymous mutations between the true DFE (light blue) and the average inferred DFE for each model of selection on synonymous mutations. Bars represent an average of 20 replicates; error bars show standard deviations. DFE bins range from neutral (0-1E-5) and nearly neutral (1E-5-1E-4) to strongly deleterious (>0.01). c) Standardized residuals of the probability mass in each DFE bin, obtained by subtracting the true probability mass (true model bars in b) from the average for each condition, for each bin, divided by the square root of the true probability mass.

**Fig. 3.**
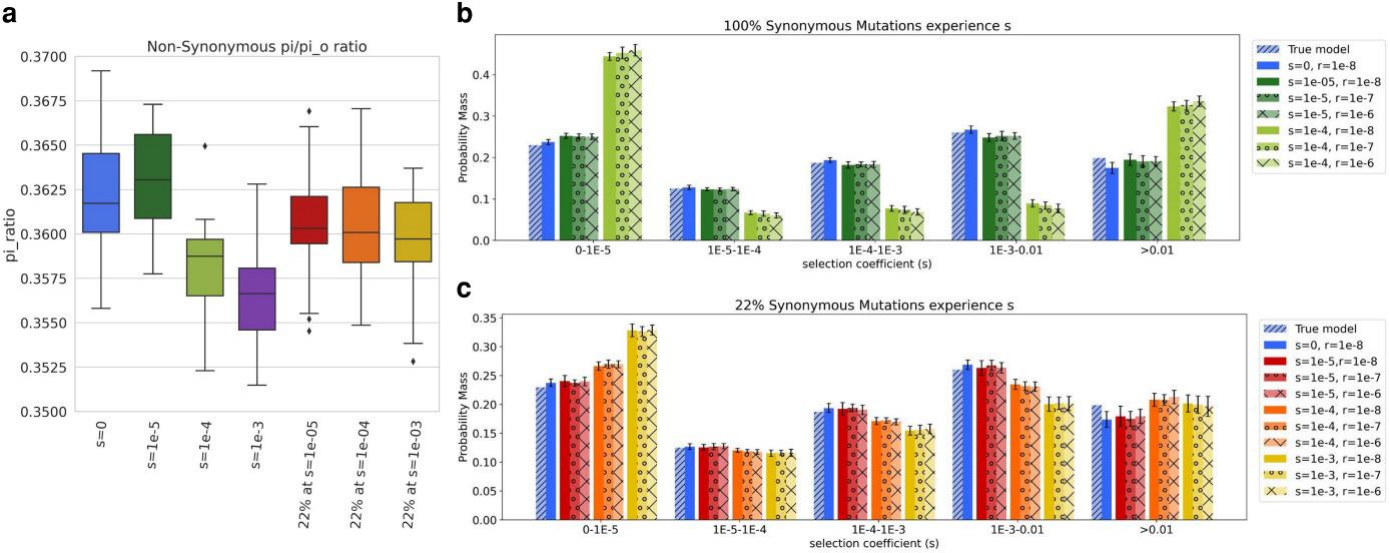
Inference of the DFE of nonsynonymous mutations under varying strengths of recombination. a) Quantification of the effects of linked selection on nonsynonymous diversity by the ratio of observed to expected number of pairwise differences, π/π_0_. For each simulation replicate, π was computed from the SFS and π_0_ was equal to 4*N*_a_*L*μ (see Methods). For each condition, the box indicates the IQR (interquartile range) of the distribution, with the horizontal line inside the box indicating the median value. Whiskers extend to the farthest point within 1.5 units of the IQR. Outliers are plotted. Each condition contains 20 replicates. b and c) Comparison of the discretized DFE for nonsynonymous mutations between the true DFE and the average inferred DFE for each model of selection on synonymous mutations. Bars represent an average of 20 replicates; error bars show the standard deviations. DFE bins range from neutral (*s* between 0-1E-5) and nearly neutral (*s* between 1E-5-1E-4) to strongly deleterious (*s* > 0.01). Lighter colors indicate increasing recombination rate, *r*. b) Discretized DFE for conditions where 100% of synonymous mutations experienced selection. c) Discretized DFE for conditions where 22% of synonymous mutations experienced selection. Shading denotes different recombination rates.

**Fig. 4.**
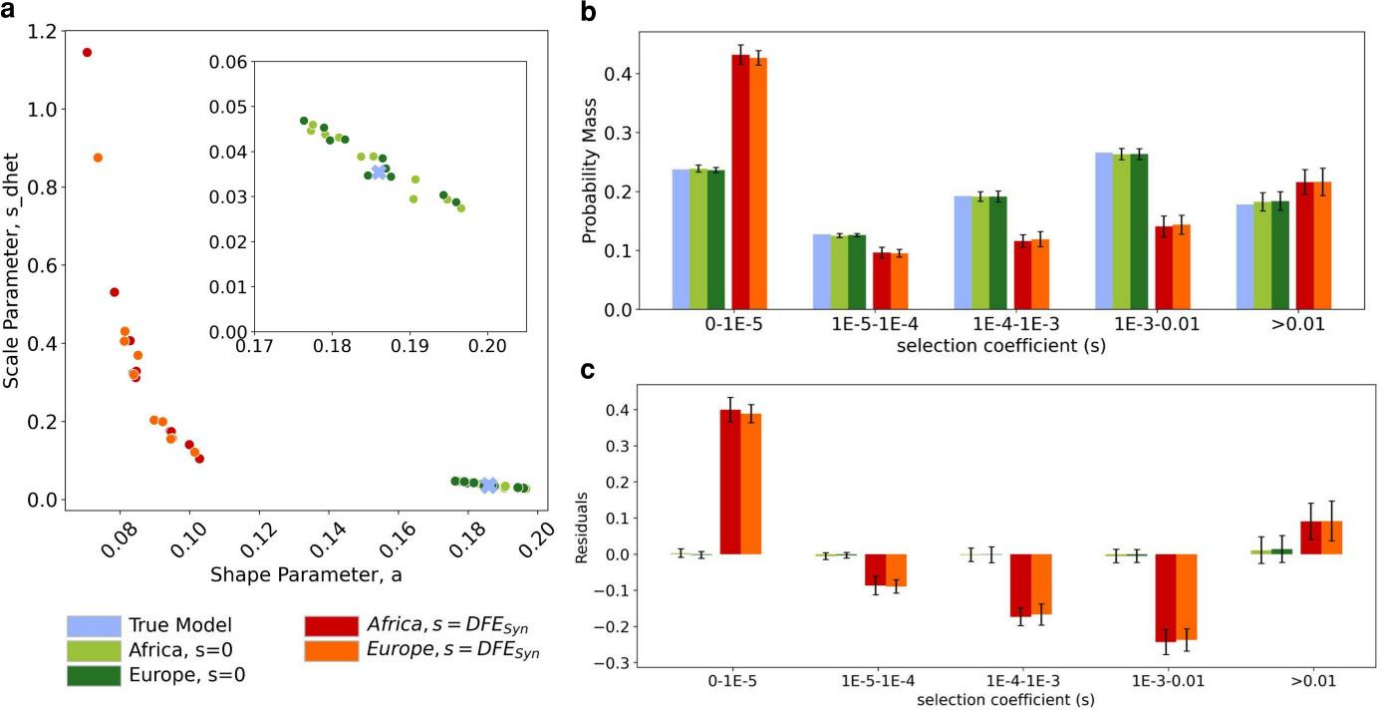
Inference of the DFE for nonsynonymous mutations under human-like parameters. Colors indicate results for simulations with and without selection on synonymous mutations. a) Inferred shape and scale parameters of a gamma DFE model for nonsynonymous mutations from simulated data with a human-like demographic history. Each point represents an individual simulation replicate. *s_dhet_* represents the scale parameter in units of heterozygous selection strength. Insert zooms in on the lower-right section of the plot. b) Comparison of the discretized DFE for nonsynonymous mutations between the true DFE (light blue) and the average inferred DFE. Bars represent an average of 10 replicates; error bars show standard deviations. DFE bins range from neutral (*s* between 0-1E-5) and nearly neutral (*s* between 1E-5-1E-4) to strongly deleterious (*s* > 0.01). c) Standardized residuals of the probability mass in each DFE bin, obtained by subtracting the true probability mass (true model bars in b) from the average for each condition, for each bin, divided by the square root of the true probability mass.

**Fig. 5.**
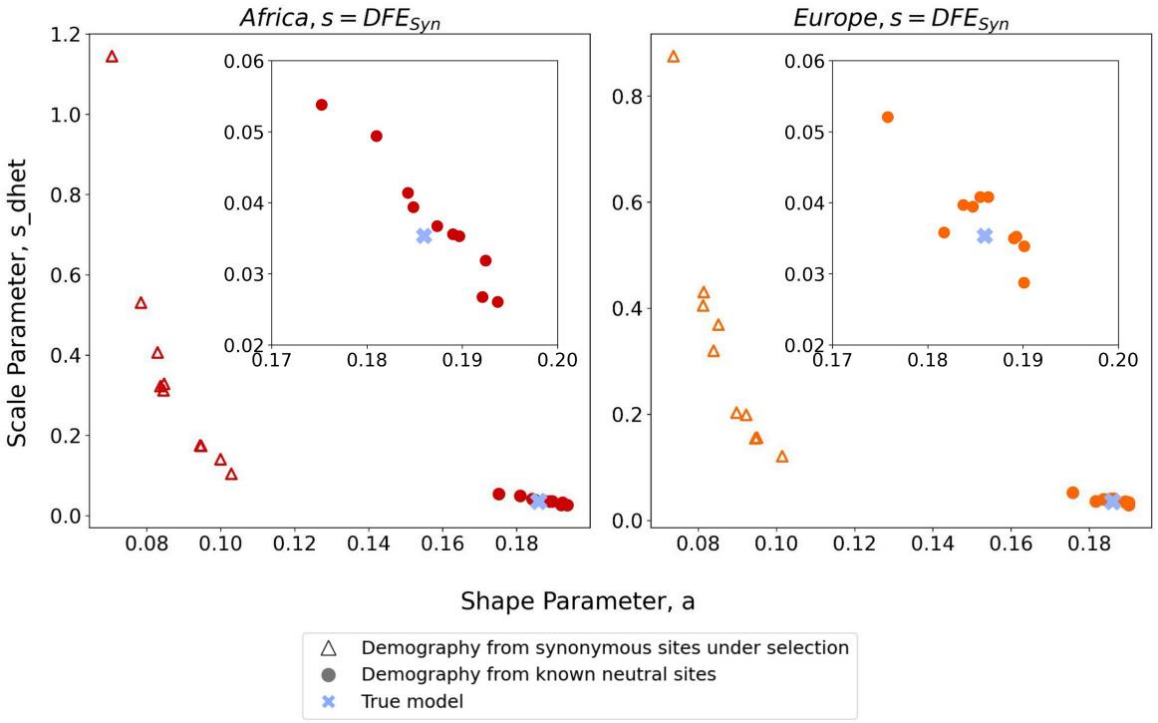
Comparison of inferences of the DFE of nonsynonymous mutations when demography was inferred from synonymous variants or a set of known neutral variants. Inferred shape and scale parameters of a gamma DFE model for nonsynonymous mutations from simulated data using human-like parameters. Each point represents an individual simulation replicate. Empty triangles represent parameter inferences generated using a demographic model inferred from synonymous variants experiencing selection, identical data as in Fig. 4a. Solid circles denote the parameter inferences generated using a demographic model inferred from truly neutral variants. Scale parameter, *s_dhet_*, represents the scale parameter in units of heterozygous selection strength.

Fit∂a∂i reports the DFE in terms of the population-scaled selection coefficient, γ = 2*N_a_s*, where *N_a_* is the ancestral population size and *s* is the selection coefficient of the heterozygote. In order to obtain the DFE in terms of *s*, we first obtained the ancestral population size for a particular replicate from the synonymous population mutation rate estimated during the demographic inference step. We use the fact that θ_s_ = 4*N_a_*μ*L*_s_, where *μ* is the per-base-pair mutation rate and *L*_s_ is the number of synonymous sites, to obtain an estimate of the ancestral population size for a given replicate. The estimation of the population size is then plugged into the formula for γ above and used to compute the scale parameter in terms of the selection coefficient of the heterozygote, *s_dhet_* = *γ/2N_a_*. Other parameters used in our study are outlined in Supplementary Table 1 for the data in Figs. 1–3 and Supplementary Table 2 for the data in Figs. 4 and 5. For more details, see Kim et al. (2017).

The estimates of discretized DFE densities in each selective coefficient bin were obtained by drawing 10,000 values from a gamma distribution for each set of best-fitting parameters. Then, we took the average across all 1,000 values for each model of selection on synonymous mutations.

## Results

### Selection on synonymous mutations results in false evidence of population expansion

We first inferred the demographic history of each simulation replicate using the SFS of synonymous variants. We fit two possible demographic models to the data: a 1-epoch model and a 2-epoch model. Although we know that the 2-epoch model is incorrect, as the data were simulated under a constant population size model, we selected these two models to represent the standard procedure when researchers perform DFE inference in a population of unknown demographic history (Boyko et al. 2008).

First, we tested a model where all synonymous mutations were deleterious with a single constant selection coefficient, which we will refer to as the “constant model.” As a control, we also performed a set of simulations with no selection on synonymous mutations. We observed a skew toward rare variants in the synonymous SFS when synonymous mutations experienced selection (Supplementary Fig. 2). The skew increased with increasing strength of selection, as expected, due to the effect of purifying selection on the SFS. The shape of the nonsynonymous SFS did not show significant changes when synonymous mutations experienced selection (Supplementary Fig. 2).

After demographic inference, the 1-epoch model was preferred in 14/20 replicates in the control simulations (*s* = 0 on all synonymous mutations). For the control simulations where the 2-epoch model was preferred (6/20), only small expansions were inferred, with at most a 1.11-fold difference between the current and ancestral population sizes (Fig. 1a; Supplementary Table 3). Inference of small expansions from simulated data without a true expansion has been previously reported (Messer and Petrov 2013; Schrider et al. 2016; Kim et al. 2017) and is due to linked selection.

In all replicates with constant selection on synonymous mutations, the 2-epoch model provided the best fit regardless of the strength of selection. This result is consistent with the synonymous SFS being skewed toward rarer variants due to negative selection. With increasing strength of negative selection on synonymous mutations, the predicted difference between the ancestral and current population sizes became larger (Fig. 1b to d and e to g). Furthermore, increasing selection led to more recent estimates of the expansion (Fig. 1 b to d and e to g). In the most extreme constant condition (*s* = 1e-3; Fig. 1d), we were unable to find a demographic model that would properly match the observed synonymous SFS. The best-fitting parameters represented the most extreme parameters values allowed in our inference (see Methods). For example, the difference between the ancestral and current population size was estimated to be on the order of 3000-fold, which led to ancestral population sizes on the order of ∼3 individuals (Fig. 1d). Interestingly, when all synonymous mutations experienced weak selection (*s* = 1e-5; Fig. 1b and e), the variance in the inferred timing and magnitude of the population expansion increased. In some replicates, large expansions were predicted, larger even than those consistently predicted in the moderately deleterious constant condition (compare Fig. 1b to c). The increased variance was accompanied by larger ranges of demographic parameters with small differences in the log-likelihood (Supplementary Fig. 3b and d), suggesting that there is limited information in the data to precisely estimate the misspecified parameters.

Next, we examined the “partial model” where only 22% of synonymous mutations were under selection (see Methods). We again observed a skew toward rarer variants only in the synonymous SFS (Supplementary Fig. 2). The skew became greater with increasing negative selection on the synonymous mutations, but overall, the effect was less pronounced in the partial model than in the constant model. For the replicates with moderate and strong selection on synonymous mutations (*s* > 1e-4, when *N* = 10,000), all showed a significantly better fit for the population expansion model compared to the 1-epoch model. For the weak selection condition (*s* = 1e-5, when *N* = 10,000), 9/20 replicates reported a 1-epoch model as the best fit to the data. However, when the 2-epoch model provided the best fit, the estimated size and timing of the expansion varied dramatically. These results were qualitatively similar to what we observed in the constant model, where weaker selection on the synonymous mutations increases the variance in the MLEs of the 2-epoch model parameters.

In summary, in nearly all of the scenarios that included selection on synonymous mutations, demographic inference showed evidence of a population expansion, even though no population size change took place.

### Inference of the DFE of nonsynonymous mutations is robust to small amounts of selection on synonymous mutations

Next, we interrogated how selection on synonymous mutations affects inference of the DFE of nonsynonymous mutations. We performed DFE inference using the software package Fit∂a∂i (Kim et al. 2017; see Methods).

From our control simulations, where synonymous mutations were neutrally evolving (dark blue dots in Fig. 2a; Supplementary Fig. 4a), we were able to recover the expected nonsynonymous DFE shape and scale parameters with only a slight underestimation (average estimates across all 20 control replicates *s_dhet_* = 0.03471 ± 0.0013; *a* = 0.1875 ± 0.0013) with respect to the true parameters (true *s_dhet_* = 0.04375; *a* = 0.186). This slight underestimate is consistent with previous results (Kim et al. 2017).

In the cases where the selective pressure on synonymous mutations was weak (*s* = 1e-05, red and dark green in Fig. 2a), DFE parameter estimates overlapped with the parameter estimates obtained in the control replicates (blue dots in Fig. 2a), indicating that weak selection on a fraction of the synonymous mutations did not impact our ability to estimate the true DFE parameters.

Only when the selection coefficient on synonymous mutations was equal or greater to *s* = 1e-4 (light green, orange, and yellow dots in Fig. 2a; Supplementary Fig. 5) were the inferred DFE parameter estimates for nonsynonymous mutations systematically distinct from true values (light blue star and dark blue dots in Fig. 2a). A deviation was observed regardless of whether selection was acting on all or a portion of synonymous variants, but was more pronounced when selection was acting on all synonymous mutations (light green dots on Fig. 2a). When all synonymous mutations were strongly selected against (*s* = 1e-3, purple lines in Fig. 1), we were unable to fit a set of DFE parameters that predicted the observed nonsynonymous SFS (see Supplementary Fig. 6).

Next, we checked whether inferences of the proportions of mutations with different selection coefficients were affected by selection on synonymous mutations. To do this, we divided the DFE into 5 bins and found the probability mass in each bin from the MLEs of the parameters of the gamma distribution (Fig. 2b and c; Supplementary Fig. 4b and c; see Methods). Encouragingly, when selection on synonymous mutations was weak (*s* = 1e-5; dark green and red bars in Fig. 2b and c) or only occurred on 22% of synonymous mutations (red, orange, and yellow), the DFE for nonsynonymous mutations appeared to be accurately inferred, with only slight biases (Fig. 2b and c). There was a slight overestimation of the proportion of neutral mutations and a slight under estimate of the proportion of moderately deleterious mutations (1e-3 < *s* < 0.01).

In the case where all synonymous mutations experienced moderate selection, we observed a larger deviation between the inferred proportions and the true proportions (light green bars in Fig. 2b and c). Here there was a large overestimation of the proportion of neutral (0-1e-5) and strongly deleterious (>0.01) nonsynonymous mutations. This overestimation was compensated by underestimating the proportion of mutations of intermediate selective strength.

We also evaluated the effect assuming different demographic models with comparable log-likelihoods on downstream DFE inferences. Overall, the estimates of the DFE parameters made when assuming different demographic models were quite similar to each other (Supplementary Fig. 7). These findings suggest that estimates of the DFE are robust to the specific demographic model selected, as long as the one chosen fits the data well.

In summary, selection on synonymous mutations affects inferences of the DFE for nonsynonymous mutations only when selection is moderate and pervasive (s ≥ 1e-4 on all mutations in a population of size *N* = 10,000). When selective pressures are moderate to weak (*s* ≤ 1e-4) or not pervasive, nonsynonymous DFE inferences are robust to unaccounted for selection on synonymous mutations.

### Decreasing linked selection through increasing recombination does not improve accuracy of DFE estimation

We next explored which factors could have biased the parameter estimates of the DFE for nonsynonymous mutations when selection acted on synonymous mutations. Linkage among sites experiencing selection impacts patterns of variation across the genome (Charlesworth et al. 1993; Fay and Wu, 2000; Cai et al. 2009; Sella et al. 2009; Hernandez et al. 2011; Lohmueller et al. 2011; Cutter and Payseur 2013; Elyashiv et al. 2016; Charlesworth and Jensen 2021; Murphy et al. 2022). In particular, background selection affects our ability to infer the demographic history of a population (Messer and Petrov 2013; Ewing and Jensen 2016; Schrider et al. 2016; Pouyet et al. 2018; Johri et al. 2021). Given the large number of synonymous mutations potentially experiencing negative selection in our simulations, we tested how linkage affects the demographic and DFE inferences.

First, to investigate the magnitude of the effects of background selection due to synonymous mutations experiencing selection, we computed the ratio of π/π_0_ for nonsynonymous and synonymous mutations (Fig. 3a; Supplementary Fig. 8; see Methods). Background selection reduces π along the genome and lowers π from its expected value under neutral evolution, π_0_ (Comeron 2014; Cvijović et al. 2018). As expected, the nonsynonymous π/π_0_ was below 1 in our control simulations, due to the selection on the nonsynonymous mutations themselves (blue box, Fig. 3a). In the scenarios where synonymous mutations were under selection, there was a small, but consistent (0.005 difference in median value between the control and most extreme constant condition, purple box), reduction in the median nonsynonymous π/π_0_. The DFE of the nonsynonymous mutations was identical across all the simulation scenarios; therefore, the reduction in the nonsynonymous π/π_0_ indicates modest, but consistent, effects of background selection when synonymous mutations were deleterious. Similar to the nonsynonymous case, the synonymous π/π_0_ was near 1 in the control simulations with no selection on synonymous mutations. However, when synonymous mutations were under selection, there was a drastic reduction in π/π_0_ with respect to the control. The reduction increased with the strength of selection on synonymous mutations, as expected, due to purifying selection decreasing the synonymous diversity (Supplementary Fig. 8).

After confirming a detectable amount of background selection on the nonsynonymous mutations due to selection on synonymous mutations, we tested whether the effects were sufficient to impact the nonsynonymous DFE inference by performing additional simulations with higher recombination rates to reduce linkage and, consequently, the effects of background selection. For the control simulations without selection on synonymous mutations (Supplementary Fig. 9), we observed similar accuracy in the inference of the DFE of nonsynonymous mutations regardless of recombination rate, indicating that recombination rate by itself does not impact DFE parameter inference. We did, however, observe an elevated number of simulations reporting a 1-epoch model as the best-fitting model for simulations with higher recombination rate (Supplementary Table 3), which is consistent with previous observations of linked selection causing mis-inferences of the demographic model (Kim et al. 2017).

Turning to the simulations with selection on synonymous mutations, we observed that the demographic inferences were closer to the true model with increased recombination in the partial model conditions. There were a greater number of replicates where the 1-epoch was selected as the best-fitting model in the condition with weak partial selection on synonymous mutations (Supplementary Table 3). However, in the partial models with weak and moderate selection on synonymous mutations (red and orange lines in Supplementary Fig. 10b), as well as in the constant model with weak selection on synonymous mutations (dark green in Supplementary Fig. 10a), the variance of the reported ancestral population size increased with increasing in recombination rate. In the strong partial selection condition (yellow lines in Supplementary Fig. 10b), the 2-epoch model still provided the best fit to all replicates, but there was a consistent increase in the reported ancestral population size (*N_a_*) of the population (Supplementary Fig. 11) toward the true simulated *N_a_*.

Increasing the recombination rate did not bring the inferred DFEs for nonsynonymous mutations closer to the underlying true DFE used in the simulations (Fig. 3). Regardless of the model of selection on synonymous mutations, there was no significant difference in the proportion of nonsynonymous mutations estimated in each selection category across the different recombination rates. This result is in line with the nonsynonymous π/π_0_ results, which showed only a slight background selection effect from the selection occurring on synonymous mutations.

In summary, increasing the recombination rate did not improve our ability to infer the DFE when synonymous mutations were experiencing selection. We therefore conclude that linkage is not a major contributing factor to the biased DFE parameter estimates under pervasive and moderate selection on synonymous mutations.

### Inference of the nonsynonymous DFE is compromised under human-like conditions by selection on synonymous mutations

Thus far, we only simulated constant-size populations. To examine a more realistic scenario, we simulated a human-like demographic history with four populations, modeling the out-of-Africa expansion described by Gravel et al. (2011)(Supplementary Table 2; Methods). We also used a deleterious DFE for synonymous mutations estimated from a sample of 797 French Canadian individuals (Ragsdale et al. 2018; Methods; Supplementary Fig. 12).

A 2-epoch demographic model is an oversimplification of the true underlying demographic history in these human-like simulations, but previous studies have shown that a 2-epoch model provides a reasonable approximation to the synonymous SFS under unknown population histories and enables robust nonsynonymous DFE inferences (Keightley and Eyre-Walker 2007; Kim et al. 2017). Our work recapitulated these results. When synonymous mutations did not experience selection, we were able to recover the original nonsynonymous DFE parameters using this simple model (Fig. 4a, green dots). When synonymous mutations experienced selection, the 2-epoch demographic model predicted a smaller and more recent population expansion (Supplementary Fig. 13) with respect to the neutral synonymous mutation case.

When selection was acting on synonymous mutations, the nonsynonymous DFE parameters were significantly skewed with respect to the true values (Fig. 4a, red and orange dots). This parameter skew translates into a large overestimation of the proportion of nearly neutral nonsynonymous mutations (Fig. 4b and c, red and orange bars). We also observed an underestimation in the other bins of the DFE, but particularly in the moderately deleterious bins (1e-4 < *s* < 0.01).

In summary, previously inferred parameters for human demography as well as a DFE for selection on synonymous mutations may bias parameter estimates of the DFE for nonsynonymous mutations.

### Using truly neutral variants improves DFE inference

As we have shown, selection on synonymous mutations can confound inference of demographic history (Fig. 1; Supplementary Fig. 13). The inference of the DFE is conditioned on the inferred demographic history of the population. We hypothesized that using the mis-inferred demographic parameters led to the biases in the inferences of the nonsynonymous DFE.

To test this idea, we assumed that there is a set of truly neutral variants that is known and can be used for demographic inference. These truly neutral variants could either be synonymous variants that have been validated to be truly neutrally evolving or some other set of noncoding polymorphisms in the genome that that could be linked or unlinked to the nonsynonymous mutations.

To simulate a scenario where the neutral variants were unlinked to the nonsynonymous mutations, we paired every nonsynonymous SFS from each simulation replicate experiencing selection on synonymous mutations with a set of demographic parameters inferred from our neutral simulations (Supplementary Fig. 14). We tested this approach in our constant population simulations as well as our human-like simulations. Since each group of simulations shared a common demographic history, the demographic parameters obtained from the neutral simulations in each group represent an estimate of the demographic parameters we would expect to obtain if we had access to a set of known neutral variants. Because we paired SFS from different simulation replicates, our known neutral variants were unlinked from the nonsynonymous variants.

When we conditioned our DFE inferences on demographic parameters obtained from known neutral variants, we were able to recover the true DFE parameters under all models of selection on synonymous mutations and all demographic histories (Fig. 4; Supplementary Fig. 15). This was reflected by the fact that the points all cluster more closely to the blue cross, which denotes the true parameter values. Furthermore, the distributions of the shape and scale parameters from the inferences where demography was inferred from truly neutral sites are indistinguishable from the DFE parameter distributions obtained from our control simulations (Supplementary Fig. 16).

In simulations with a constant population size, the conditions with weak selection on synonymous mutations (*s* = 1e-5) are the only conditions where there is little difference between the parameters inferred using the standard pipeline vs the truly neutral demographic history (Supplementary Fig. 15, dark green and red dots). Using truly neutral variants also allowed us to recover the DFE parameters in the case where selection on synonymous mutations was strongest (*s* = 1e-3; Supplementary Fig. 15, purple triangles), which we were previously unable to infer due to the lack of convergence of extreme demographic parameters predicted by the synonymous mutations. Under a human-like demography, we were able to recover the DFE parameters (Fig. 4, orange and red dots) with comparable accuracy to the control simulations (Fig. 3a, green dots).

In summary, conditioning DFE inferences on demographic parameters inferred from a set of truly neutral variants allowed us to recover the true DFE parameters for nonsynonymous mutations, even when the truly neutral variants were unlinked from the nonsynonymous mutations. This result provides further understanding as to how selection on synonymous mutations confounds inference of the nonsynonymous DFE. Specifically, selection on synonymous mutations leads to misspecification of the demographic history when inferring the DFE for nonsynonymous mutations.

## Discussion

In this work, we investigated the effects of selection on synonymous mutations on SFS-based methods for estimating demographic history and the DFE of nonsynonymous mutations. We made use of forward-in-time simulations to show the impact of varying levels of selective pressure on synonymous mutations when conducting evolutionary inferences. As previously suggested (Ragsdale et al. 2018), we found that inferring demographic parameters from synonymous variants experiencing selection leads to false estimates of population expansions. The inferred magnitude of the expansion (ratio between the current and ancestral population sizes) increased with increasing negative selection on synonymous mutations. When considering the DFE parameters, performance depended on the strength of selection and the amount of synonymous variants experiencing selection.

More specifically, if the population size is constant over time, DFE inferences are only impacted when selection on synonymous mutations is pervasive and moderate (*s* ≥ 1e-4 on all mutations, when *N* = 10,000). The deviations from the true parameters resulted in overestimation of the proportion of nearly neutral and strongly deleterious mutations expected in the population. Under human-like parameters for demographic history and strength of selection on synonymous mutations, DFE inferences deviated from the true parameters. We found a large overestimate of the proportion of nearly neutral mutations, an effect that is consistent with the results observed in constant-size populations. This can be understood by noticing that the gamma-distributed DFE for synonymous mutations used in the human-like simulations (Supplementary Fig. 12; Ragsdale et al. 2018) included strong selection on synonymous mutations. Namely, in this model, ∼30% of synonymous mutations have a selection coefficient ≥1e-4 and ∼10% have a coefficient >1e-3, which most closely resembles the constant-size population cases with pervasive and moderate selection on synonymous mutations (*s* ≥ 1e-4 on all mutations). We showed that the deviations in the nonsynonymous DFE inferences are primarily mediated by conditioning on incorrect demographic parameter estimates obtained from synonymous variation.

The excess of neutral and strongly deleterious mutations inferred in the nonsynonymous DFE can be understood by realizing the interplay between the demographic correction and the shape of the nonsynonymous SFS. The selection on synonymous mutations skews the synonymous SFS toward rare variants. Since the purifying selection on synonymous variants is not accounted for, ∂a∂i interprets the excess of rare variants as a demographic effect, in particular, an expansion. The stronger the selection on the synonymous mutations, the more recent and larger the predicted population expansion is to account for the depletion of common synonymous variants. Then, DFE inference for nonsynonymous mutations conditions on this demographic model, which predicts a high proportion of rare variants. When using such an expansion model to infer the nonsynonymous DFE parameters, the nonsynonymous variants at low frequency are accounted for by the demographic expansion, and therefore selection is not required to explain these variants. This leads to an overestimation of the proportion of neutral and strongly deleterious mutations. Of note, strongly deleterious mutations are not expected to be segregating in the sample and are not contributing to the skew in the SFS.

Our study points to a potential solution to inferring demography and the DFE in the presence of selection on synonymous mutations. Specifically, the main contributor to the inaccuracy in the inferred DFE parameters of nonsynonymous mutations is the use of incorrect demographic parameters (Fig. 5). We showed that using a demographic model inferred from known neutral variants improved the fit and accuracy of the inferred DFE parameters. Hence, one solution would be to use truly neutrally evolving sites for demographic inference instead of synonymous variants. For example, a potential approach consists of using short intronic variation to obtain a neutral SFS from which the demographic history of the population can be inferred. Previous studies in *Drosophila* have shown that variants in short introns, defined as introns less than 65 bp, experience little selection in *Drosophila*, particularly between the 8 and 30 bp positions (Parsch et al. 2010; Clemente and Vogl 2012). Several studies have made use of short introns as a neutral reference, but a systematic approach has not yet been developed (Eyre-Walker and Keightley 2009; Lawrie et al. 2013; Racimo and Schraiber 2014). Another possible source of known neutral variation consists of ancestral transposable elements, although their use has only been proposed in mammals (Lunter et al. 2006). When an annotated genome for the organism of interest is available, such as is the case for humans, it is possible to use putatively neutral regions flanking coding sequences to match the expected effects of linked selection, as demonstrated by Huang and Siepel (2019). Finally, some studies have also used intragenic variants from putatively neutral regions as a reference (Gazave et al. 2014; Ragsdale et al. 2018). Ragsdale et al. compared the DFE of nonsynonymous variants obtained from using synonymous vs intragenic variants as the putative neutral variants from which to infer demography and found an increase in the estimated proportion of strongly selected mutations (*s* ≥ 1e-2) from 19% to 22%.

One main advantage to using synonymous variants for demographic inference is that they are interdigitated with the nonsynonymous mutations and thus provide an approximate control for the effects of linked selection, which affects both synonymous and nonsynonymous mutations (Kim et al. 2017). If the short introns used for demographic inference are chosen to be located nearby the exons, then they may still be sufficiently linked to the nonsynonymous mutations to account for the background selection effect, which would represent an advantage over using intragenic variants or transposable elements. Because linked neutral variants have been such a successful control for the effects of linked selection (Kim et al. 2017), we were concerned that unlinked neutral variants would not adequately control for background selection. However, the degree of background selection in our simulated data using an average recombination rate (*r* = 1e-8) was not sufficient to appreciably bias our inferences of the nonsynonymous DFE, even when the neutral control variants to infer demography were unlinked to the nonsynonymous mutations (Fig. 4). Of course, in real data background selection could be stronger than what was modeled here or there could be other types of linked selection. Thus, it would also be worthwhile to investigate how the SFS varies with recombination rate.

If one has access to a known set of neutrally evolving variants, one potential test for selection acting on synonymous mutations could be to use ∂a∂i to compare the demographic parameters estimated from a set of neutrally evolving variants to the parameters estimated from synonymous variants. If the parameters differ significantly, that would suggest that selection might be impacting the synonymous variation in the dataset. Another test for selection on synonymous variants could compare the fit of a simple demographic model to one also including selection. This approach holds more promise with extremely large sample sizes (>1,000 individuals), as (incorrect) models only including demography seem to fit SFS from smaller numbers of individuals quite well.

Interestingly, we were unable to find a well-fitting demographic model for our data under conditions of pervasive and strong selection on all synonymous mutations. In particular, the inference reached the biologically informed parameter bounds, which were set to an expansion that was nearly impossible. This result suggests another potential check for selection on synonymous mutations—if the inference of demographic parameters from a natural population is proving difficult and leading to biologically nonsensical expansions, researchers should consider the possibility that there might be pervasive selection acting on synonymous mutations. In such scenarios, we would strongly recommend seeking an alternative source of neutrally evolving variants.

Our study focuses on the impact of negative selection synonymous mutations on the inference of the strength of selection on deleterious nonsynonymous mutations. However, estimates of positive selection on nonsynonymous mutations that rely on synonymous variation as a neutral control also might be impacted. For example, estimates of the proportion of substitutions that are adaptive, α, obtained from extensions of the McDonald–Kreitman test (Smith and Eyre-Walker 2002) rely on measurements of the nonsynonymous to synonymous divergence ratio with respect to the ratio observed in polymorphism data. Negative selection on synonymous mutations would reduce the observed synonymous divergence and, in the scenario where selection on synonymous mutations is strong, also reduce synonymous polymorphism. If the synonymous divergence is reduced relative to the amount of synonymous polymorphism, that could lead to inflated estimates of adaptation at nonsynonymous fixations. On the other hand, some analyses suggest estimates from MK-derived methods are robust to selection on synonymous variation (Uricchio et al. 2019). More work is required to fully characterize the impact on inference of adaptive evolution when synonymous mutations experience selection.

Our study has some limitations. We tested various models of selection on synonymous mutations trying to use the most up-to-date parameter estimates, but currently, there is not a well-established consensus on the synonymous DFE for most organisms. Furthermore, while it has been established that the DFE of nonsynonymous mutations is well represented by a gamma distribution (Eyre-Walker and Keightley 2007; Kim et al. 2017; Wade et al. 2023), the shape of the DFE of synonymous mutations has not been well characterized. Some studies have shown evidence that at least a portion of synonymous mutations are under moderate (*Ns* > 1) to strong (*Ns* > 10) selection in both *Drosophila* (Lawrie et al. 2013) and humans (Keightley and Halligan 2011), reporting similar proportions (∼20%) of synonymous mutations under selection in both cases. Other studies are inconsistent with the 20% estimate in humans and report more widespread selection on synonymous mutations with up to 30% of synonymous variants under moderate to strong selections (Ragsdale et al. 2018; Huang et al. 2019). Models with pervasive strong selection on synonymous mutations, as suggested by Ragsdale et al (2018), suggest selection on synonymous mutations affects DFE inference for nonsynonymous mutations. However, models with less selection, like those suggested by Keightley and Halligan and Lawrie, predict less of an effect. Thus, our simulations are meant to provide a benchmark for the effects of selection on synonymous mutations using current inference methods. The most extreme parameter conditions (all sites experiencing *s* ≥ 1e-4, when *N* = 10,000) represent edge cases and are more of a theoretical investigation than our true expectation of what the selection on synonymous variants would be in real organisms. More research is needed to better understand the distribution of fitness effects of synonymous mutations, which will in turn allow better characterization of the impact on inference methods.

Our work tests how violating the long-held assumption that synonymous mutations are neutrally evolving impacts the ability to infer demographic histories and DFE parameters of nonsynonymous mutations from genetic variation data. We demonstrated that under weak selection on synonymous variants, the impact on the inference of nonsynonymous DFE parameters is not significant. However, in organisms where selection on synonymous mutations are more substantial, the inferred DFE inferences may be biased. At this time, we do not know which model of the DFE of synonymous mutations is most accurate. Fundamentally, our results underscore the importance of better understanding the selective pressures impacting synonymous mutations in any organism.

## Supplementary Material

iyaf200_Supplementary_Data

## Data Availability

Scripts for simulations and inferences using ∂a∂i and Fit∂a∂i are located at https://github.com/amzurita/Synonymous_Selection_Project/tree/main. Supplemental material available at *GENETICS* online.
