# Supplementary material for: The impact of non-neutral synonymous mutations when inferring selection on nonsynonymous mutations
Source: Genetics. 2025 Sep 27;231(4):iyaf200. doi: 10.1093/genetics/iyaf200 (PMC12693584; doi:10.1093/genetics/iyaf200)
Supplement: iyaf200_Supplementary_Data [file iyaf200_supplementary_data.zip › Supplementary_Figure_10_GENETICS-2025-308515.docx]

**
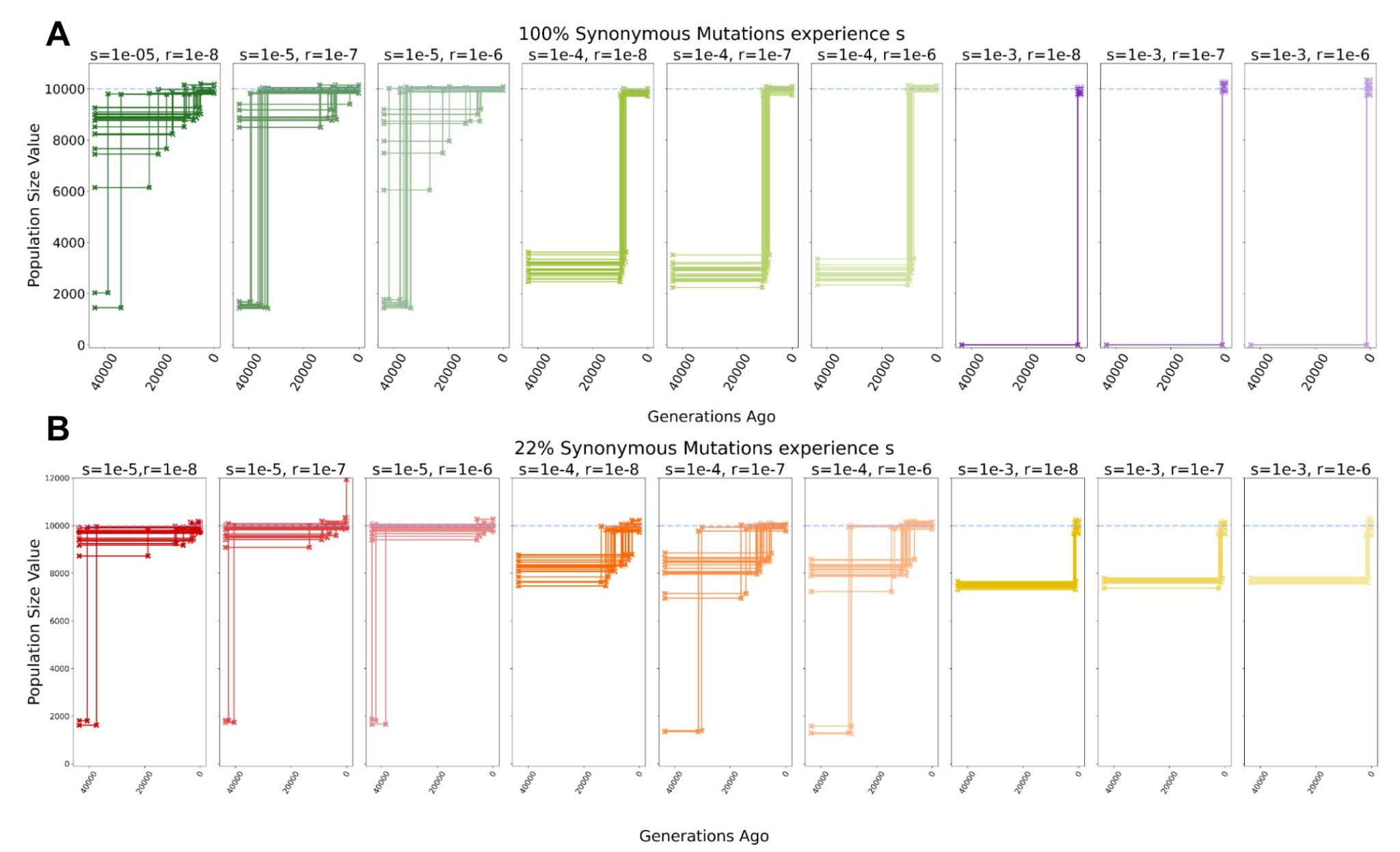
**

**Supplementary Figure 10: Inference of demography with varying degrees of selection on synonyms mutations and varying recombination rates.** Inferred population size for each replicate under each model of selection on synonymous mutations and recombination rate. Each scenario includes 20 simulation replicates. When a Two Epoch model (one population size change at a specific time in the past) provided the best fit, the inferred time of the demographic event is indicated by a step in the plot between the ancestral population size and current population size. A horizontal line indicates data best described by a One Epoch (constant population size) model. The dashed blue line corresponds to the true population size in all simulations (*N*=10000). Inference in each replicate was performed on a sample of 100 chromosomes. **A** Demographic inference for models of selection on synonymous sites where 100% of synonymous mutations experience selection. Specific selection coefficients and recombination rates are indicated by *s* and *r*, respectively, at the top of each plot. **B** Demographic inference for models of selection on synonymous sites where 22% of synonymous mutations experience the selection coefficient. Specific selection coefficients and recombination rates are indicated by *s* and *r*, respectively, at the top of each plot.
