# Supplementary material for: The impact of non-neutral synonymous mutations when inferring selection on nonsynonymous mutations
Source: Genetics. 2025 Sep 27;231(4):iyaf200. doi: 10.1093/genetics/iyaf200 (PMC12693584; doi:10.1093/genetics/iyaf200)
Supplement: iyaf200_Supplementary_Data [file iyaf200_supplementary_data.zip › Supplementary_Figure_11_GENETICS-2025-308515.docx]

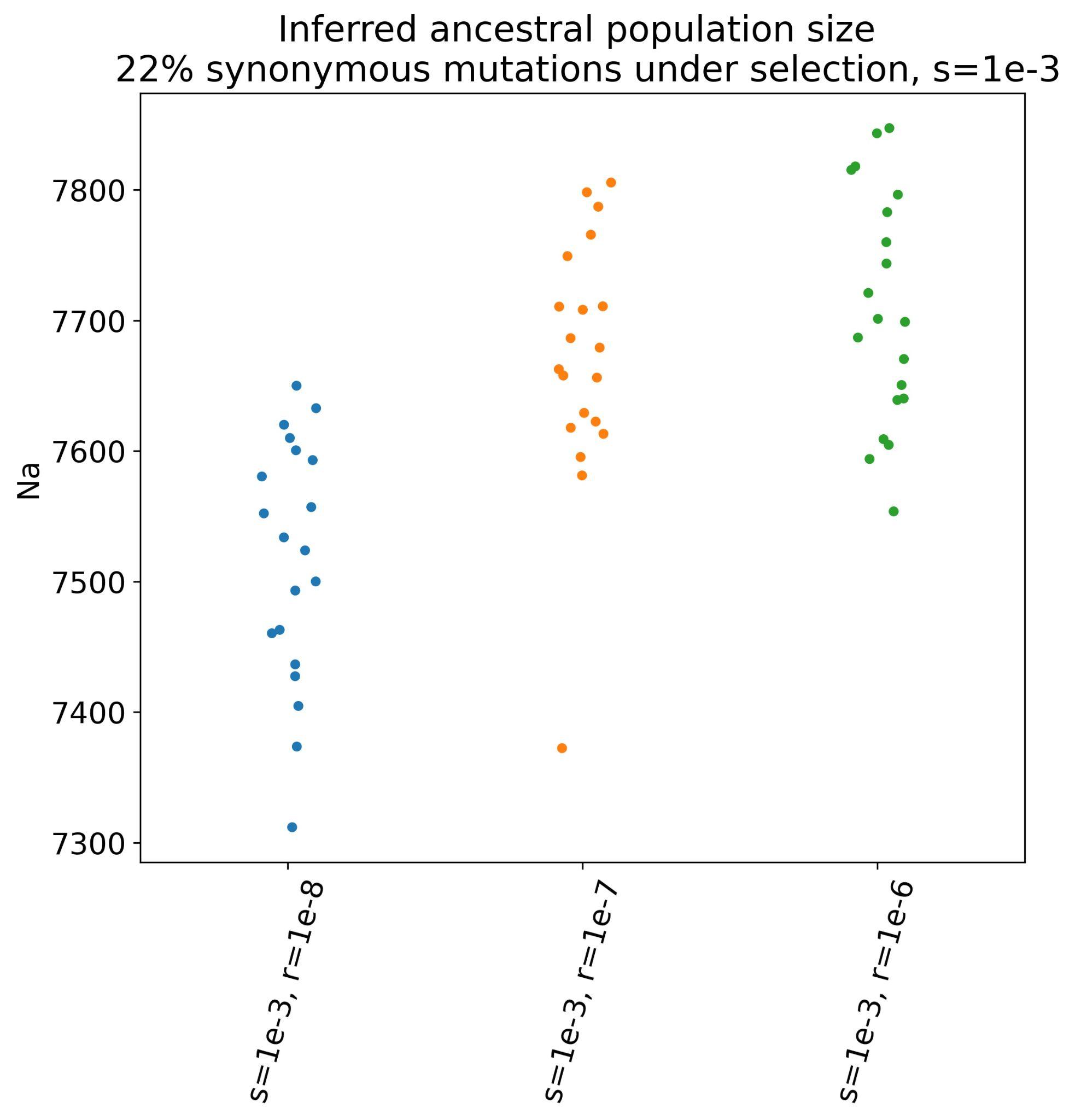


**Supplementary Figure 11: Inferred ancestral population size for replicates with 22% of synonymous mutations experiencing a selection coefficient of *s*=1e-3 with increasing recombination rate, *r***. Each dot represents an individual simulation replicate.
