# Supplementary material for: The impact of non-neutral synonymous mutations when inferring selection on nonsynonymous mutations
Source: Genetics. 2025 Sep 27;231(4):iyaf200. doi: 10.1093/genetics/iyaf200 (PMC12693584; doi:10.1093/genetics/iyaf200)
Supplement: iyaf200_Supplementary_Data [file iyaf200_supplementary_data.zip › Supplementary_Figure_12_GENETICS-2025-308515.docx]

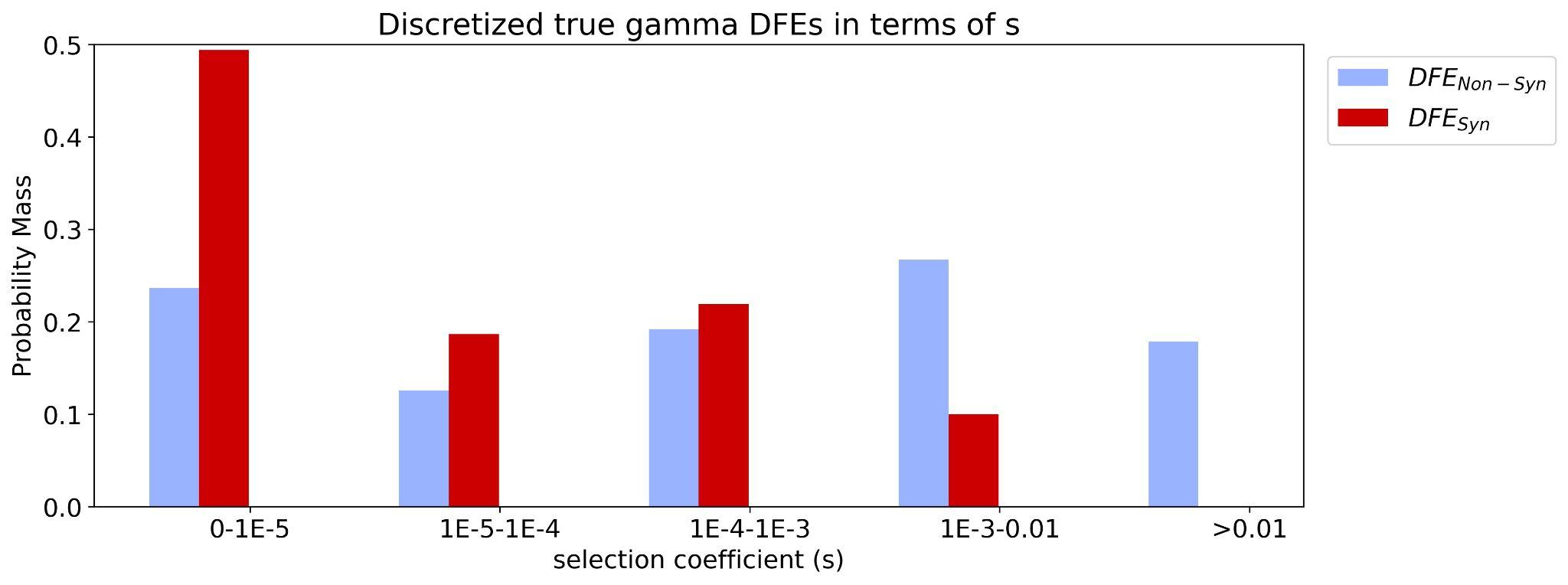


**Supplementary Figure 12: True binned distribution of fitness effects (DFE) used for human-like simulations.** Light blue indicates the true DFE for non-synonymous mutations computed from the parameters in Kim et al. 2017. Red indicates the true DFE for synonymous mutations computed with the parameters in Ragsdale et al. 2018. DFE bins range from neutral (0-1E-5) and nearly neutral (1E-5-1E-4) to strongly deleterious (>0.01).
