# Supplementary material for: The impact of non-neutral synonymous mutations when inferring selection on nonsynonymous mutations
Source: Genetics. 2025 Sep 27;231(4):iyaf200. doi: 10.1093/genetics/iyaf200 (PMC12693584; doi:10.1093/genetics/iyaf200)
Supplement: iyaf200_Supplementary_Data [file iyaf200_supplementary_data.zip › Supplementary_Figure_13_GENETICS-2025-308515.docx]

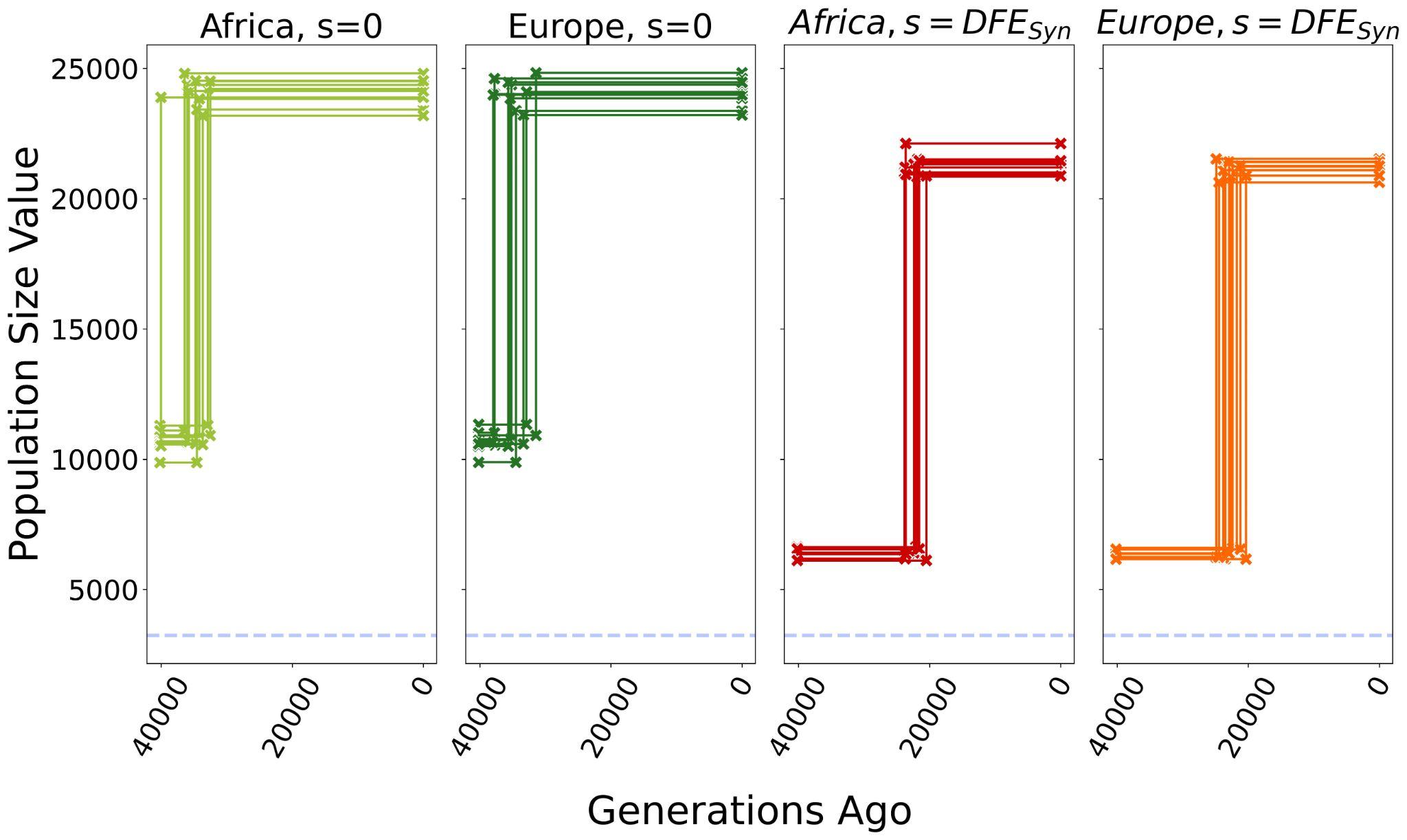


**Supplementary Figure 13: Inference of a two-epoch demographic model in human-like simulations.** Inferred population size for each replicate in the African and European populations under each model of selection on synonymous mutations. Each panel includes 10 simulation replicates. The dashed blue line corresponds to the true ancestral population size in all simulations before splitting (*N*=11293). Inference in each replicate was performed on a sample of 100 chromosomes. Presence or absence of selection on synonymous mutations is indicated at the top of each plot.
