# Supplementary material for: The impact of non-neutral synonymous mutations when inferring selection on nonsynonymous mutations
Source: Genetics. 2025 Sep 27;231(4):iyaf200. doi: 10.1093/genetics/iyaf200 (PMC12693584; doi:10.1093/genetics/iyaf200)
Supplement: iyaf200_Supplementary_Data [file iyaf200_supplementary_data.zip › Supplementary_Figure_14_GENETICS-2025-308515.docx]

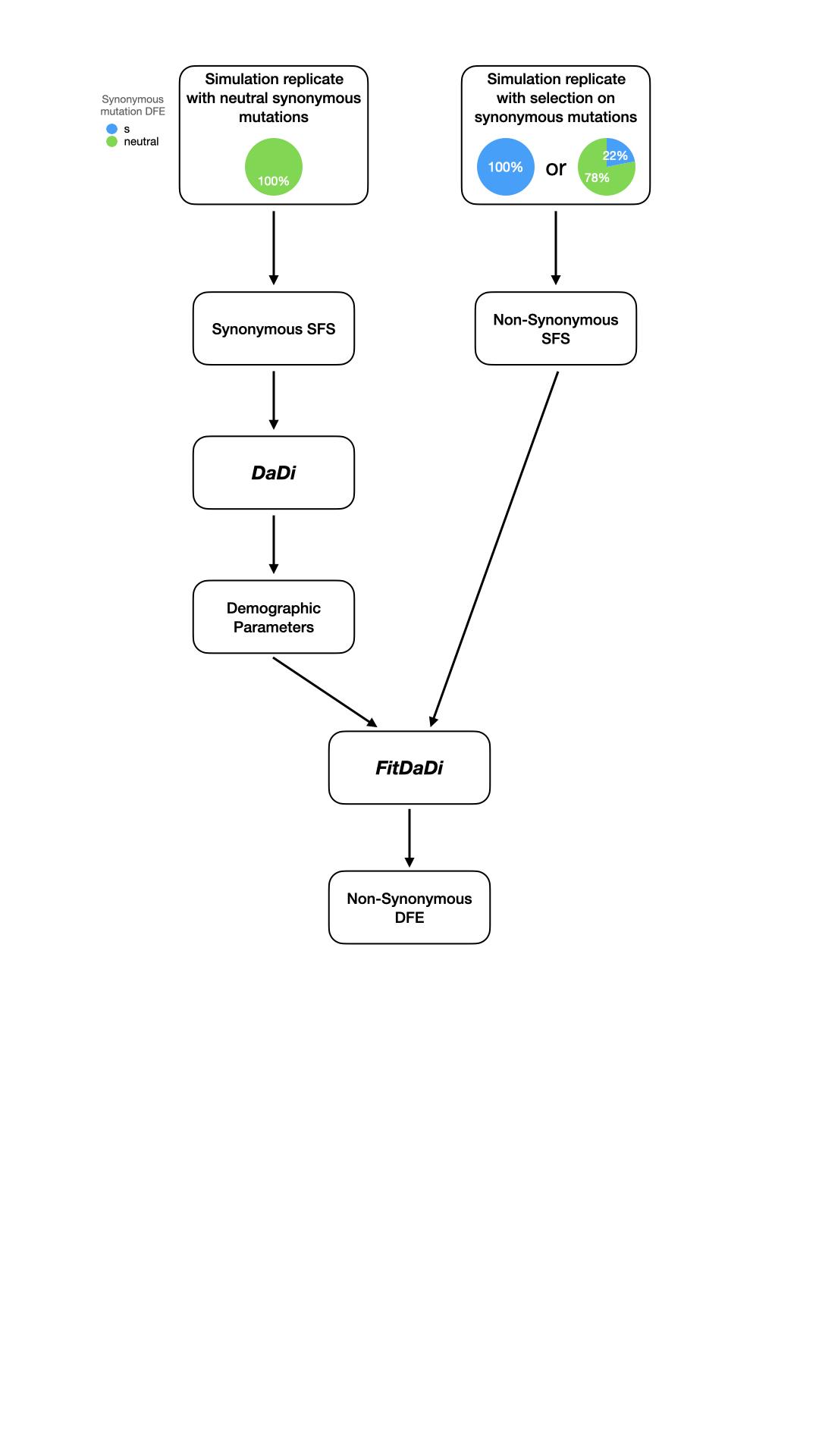


**Supplementary Figure 14: Schematic of the procedure followed to test the usefulness of a set of known unlinked neutral variants when inferring the DFE of nonsynonymous mutations.** The nonsynonymous SFS of a single replicate from a simulation where synonymous mutations experienced selection (right side) was paired with the demographic parameters obtained from a simulation replicate without selection acting on the synonymous mutations (left side). Fit∂a∂i performed the inference of the DFE of nonsynonymous mutations conditioned on the demographic parameters inferred from known neutral sites. For each of the 20 replicates simulated under each model of selection on synonymous mutations, we paired the replicate with a set of demographic parameters inferred from a simulation where synonymous mutations were entirely neutral. We conditioned the DFE inference on the inferred demographic model. For example, in replicate 1 of the constant simulation with *s*=1e-5, we conditioned the DFE inference on the demographic parameters inferred from replicate 1 of the control simulations, where *s*=0 on all synonymous mutations. Nonsynonymous mutations in all simulations have the same DFE (See Methods).
