# Supplementary material for: The impact of non-neutral synonymous mutations when inferring selection on nonsynonymous mutations
Source: Genetics. 2025 Sep 27;231(4):iyaf200. doi: 10.1093/genetics/iyaf200 (PMC12693584; doi:10.1093/genetics/iyaf200)
Supplement: iyaf200_Supplementary_Data [file iyaf200_supplementary_data.zip › Supplementary_Figure_15_GENETICS-2025-308515.docx]

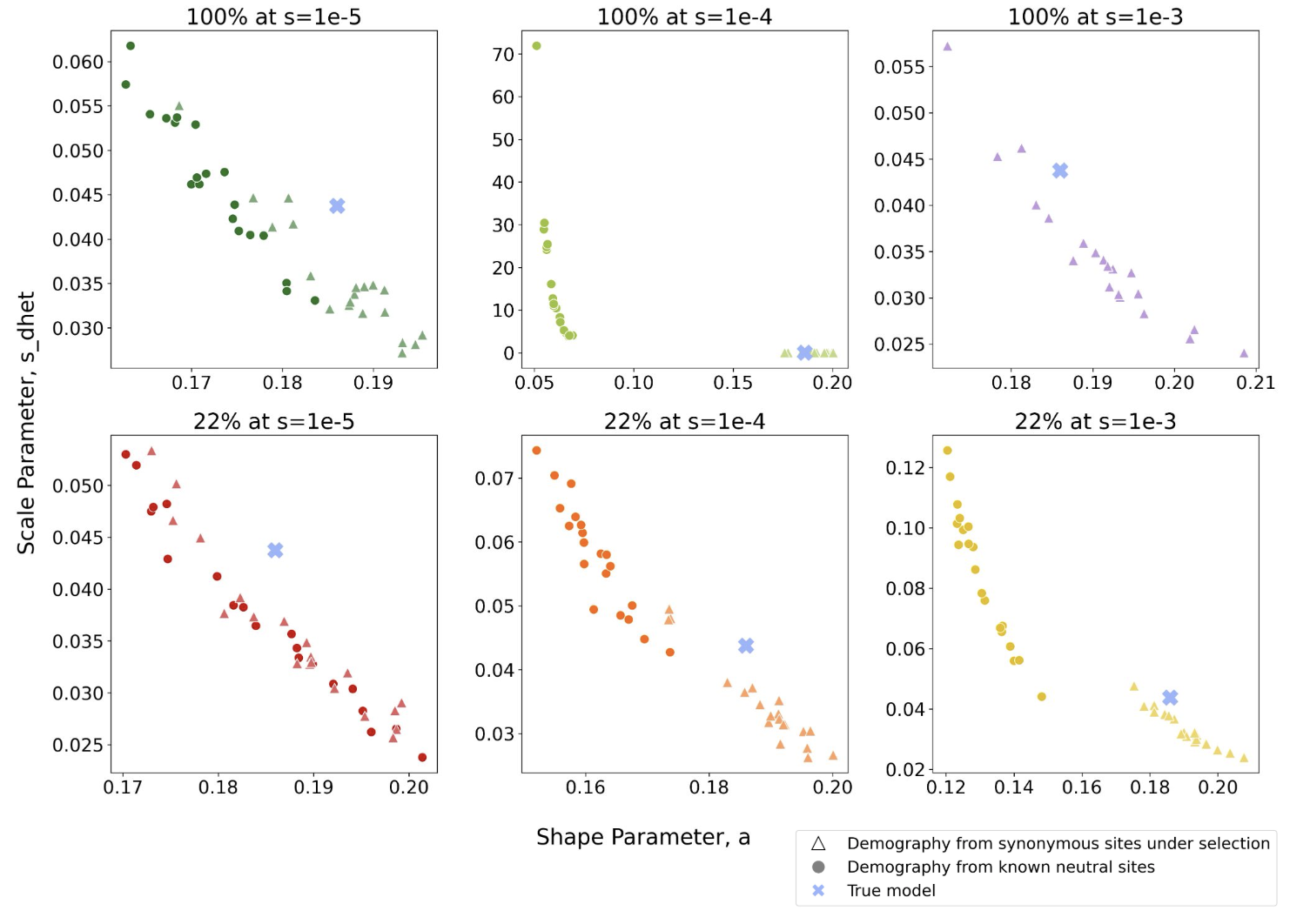


**Supplementary Figure 15: Comparison of inferences of the DFE of nonsynonymous mutations with and without demography inferred from a set of known neutral variants**. Inferred shape and scale parameters in a gamma DFE model for nonsynonymous mutations from simulated data with distinct levels of selection on synonymous mutations and a constant population size. Each point represents an individual simulation replicate. Empty triangles represent parameter inferences generated using a demographic model inferred from a synonymous SFS experiencing selection, identical data as Figure 2A. Filled circles represent parameter inferences generated using a demographic model inferred from known neutral variants. Scale parameter, *s_dhet_*, represents the scale parameter in units of heterozygous selection strength. Each plot shows inferred parameters for a distinct model of synonymous selection: **(A-C)** constant model where all mutations are affected by selection; **(D-F)** partial model of selection, where 22% of the mutations are under selection. Inserts zoom into points around true DFE parameters used to simulate the data, indicated by a blue cross.
