# Supplementary material for: The impact of non-neutral synonymous mutations when inferring selection on nonsynonymous mutations
Source: Genetics. 2025 Sep 27;231(4):iyaf200. doi: 10.1093/genetics/iyaf200 (PMC12693584; doi:10.1093/genetics/iyaf200)
Supplement: iyaf200_Supplementary_Data [file iyaf200_supplementary_data.zip › Supplementary_Figure_16_GENETICS-2025-308515.docx]

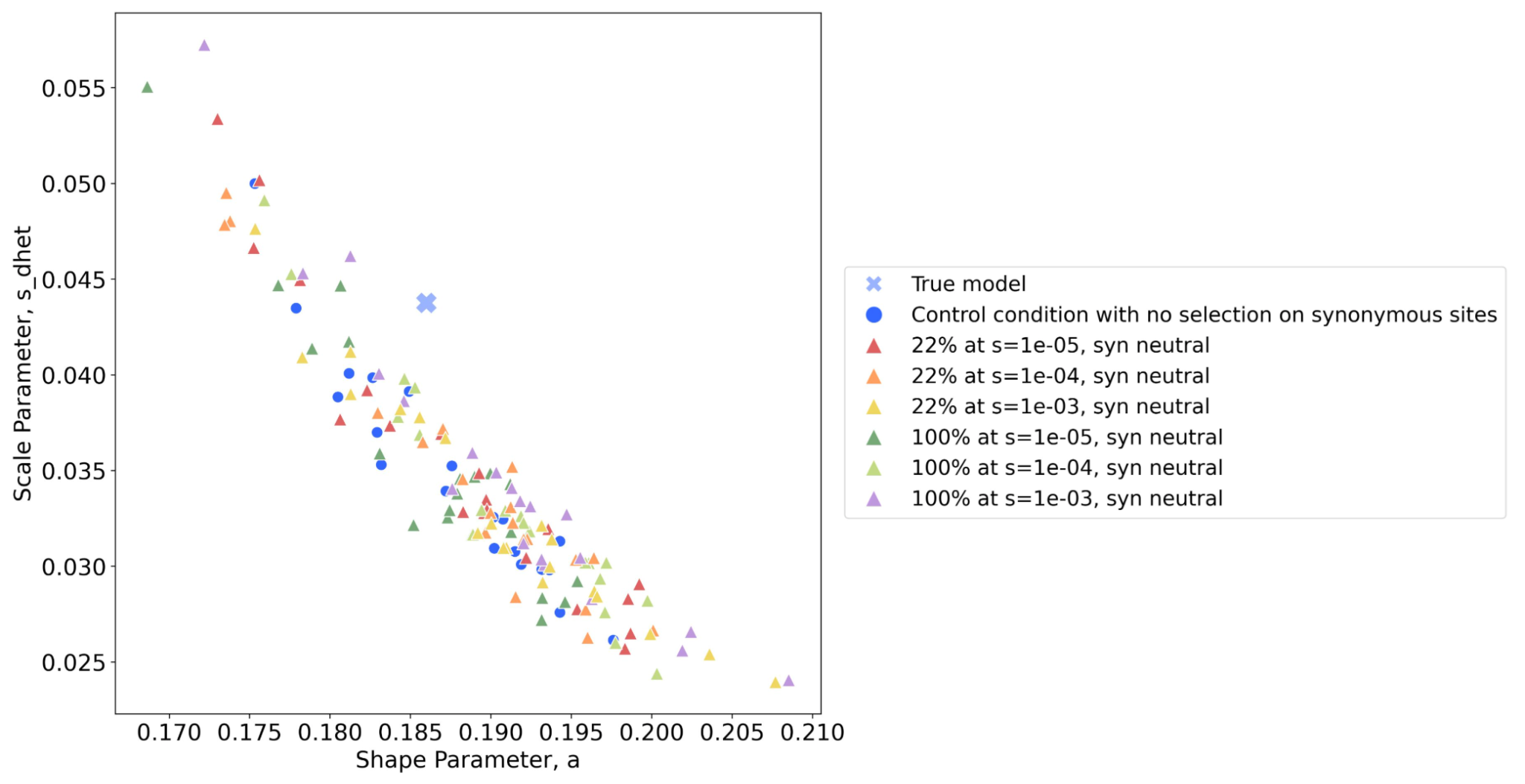


**Supplementary Figure 16: Distribution of inferred shape and scale parameters in a gamma DFE model for nonsynonymous mutations and a constant population size when demography was inferred from a set of known neutral variants.** Each point represents an individual simulation replicate. Scale parameter, *s_dhet_*, represents the scale parameter in units of heterozygous selection strength.
