# Supplementary material for: The impact of non-neutral synonymous mutations when inferring selection on nonsynonymous mutations
Source: Genetics. 2025 Sep 27;231(4):iyaf200. doi: 10.1093/genetics/iyaf200 (PMC12693584; doi:10.1093/genetics/iyaf200)
Supplement: iyaf200_Supplementary_Data [file iyaf200_supplementary_data.zip › Supplementary_Figure_1_GENETICS-2025-308515.docx]

#
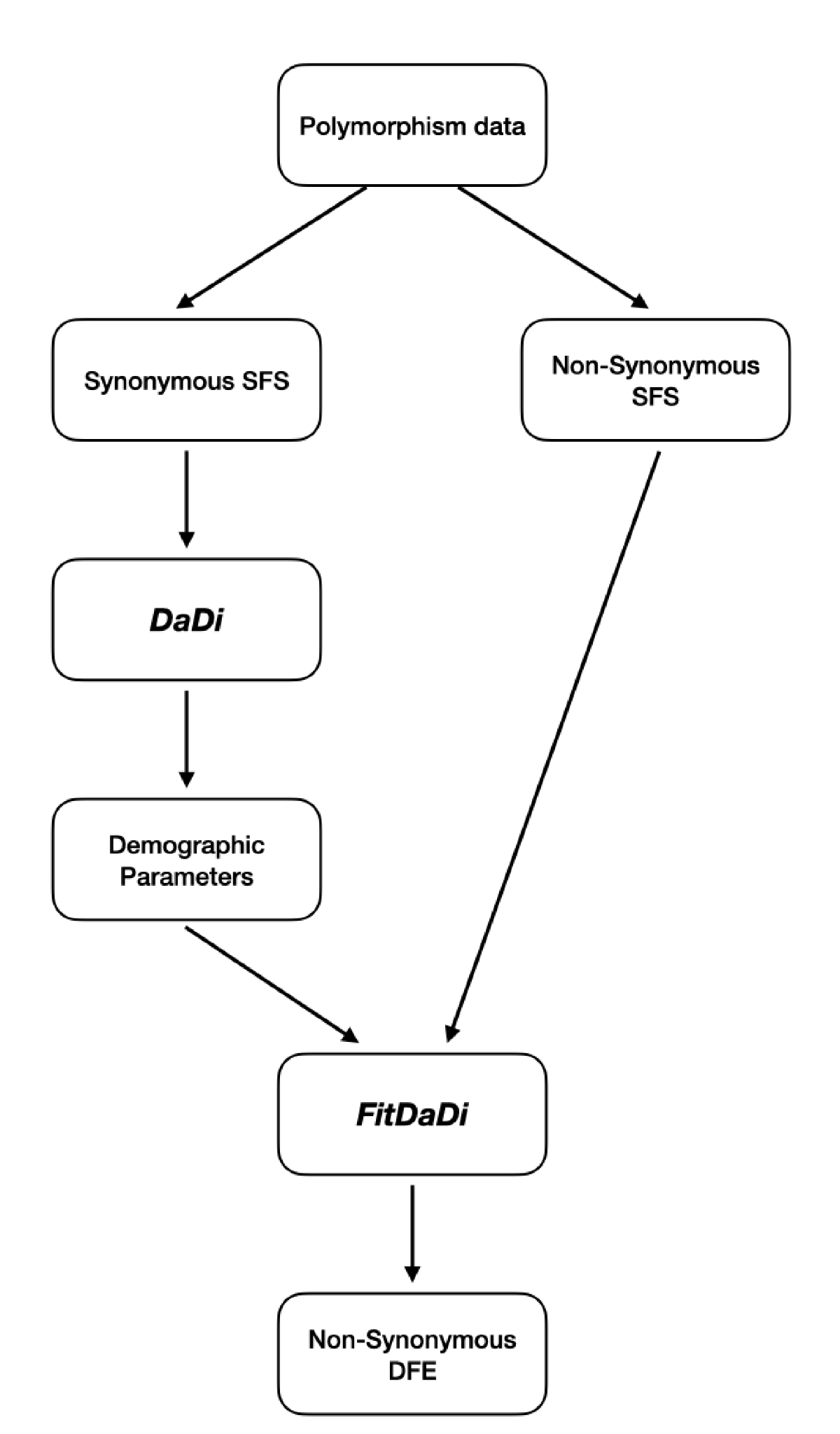


**Supplementary Figure 1: Schematic of the workflow used to infer the parameters of the distribution of fitness effects of non-synonymous mutations from polymorphism data**. The bolded and italicized words represent specific software: ∂a∂i (Gutenkunst et al. 2009) is a demographic inference software that uses a diffusion approximation approach to compute the expected SFS under a particular demographic model. Fit∂a∂i (Kim et al. 2017) is a software package that does rapid inference of DFE parameters of new mutations.
