# Supplementary material for: The impact of non-neutral synonymous mutations when inferring selection on nonsynonymous mutations
Source: Genetics. 2025 Sep 27;231(4):iyaf200. doi: 10.1093/genetics/iyaf200 (PMC12693584; doi:10.1093/genetics/iyaf200)
Supplement: iyaf200_Supplementary_Data [file iyaf200_supplementary_data.zip › Supplementary_Figure_2_GENETICS-2025-308515.docx]

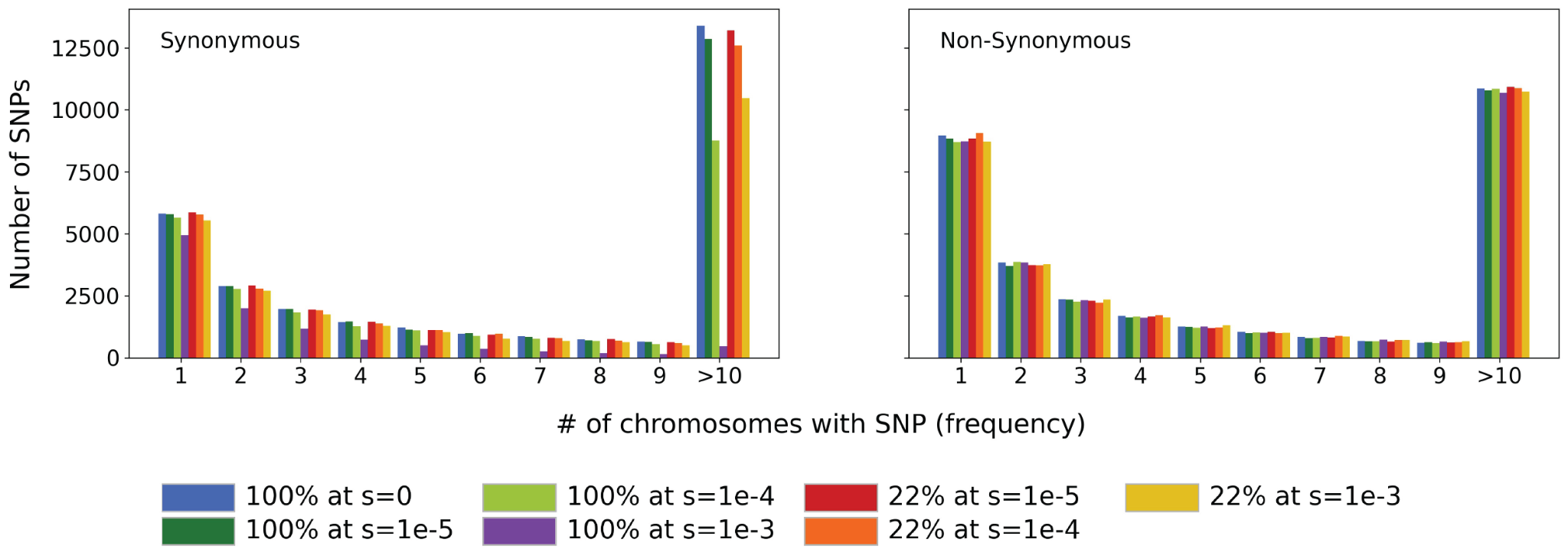


**Supplementary Figure 2: Comparison of SFS for synonymous and non-synonymous variants obtained from a single simulation replicate across models of selection on synonymous mutations**. The color of the bar indicates the specific model used in the simulation. The x-axis represents the number of chromosomes in the sample with the particular variant. Variants that are present in more than 10 individuals are summed and shown in the >10 bin, to make the visual comparison easier. The y-axis shows the number of single nucleotide polymorphisms (SNPs) found at a particular count in the sample.
