# Supplementary material for: The impact of non-neutral synonymous mutations when inferring selection on nonsynonymous mutations
Source: Genetics. 2025 Sep 27;231(4):iyaf200. doi: 10.1093/genetics/iyaf200 (PMC12693584; doi:10.1093/genetics/iyaf200)
Supplement: iyaf200_Supplementary_Data [file iyaf200_supplementary_data.zip › Supplementary_Figure_3_GENETICS-2025-308515.docx]

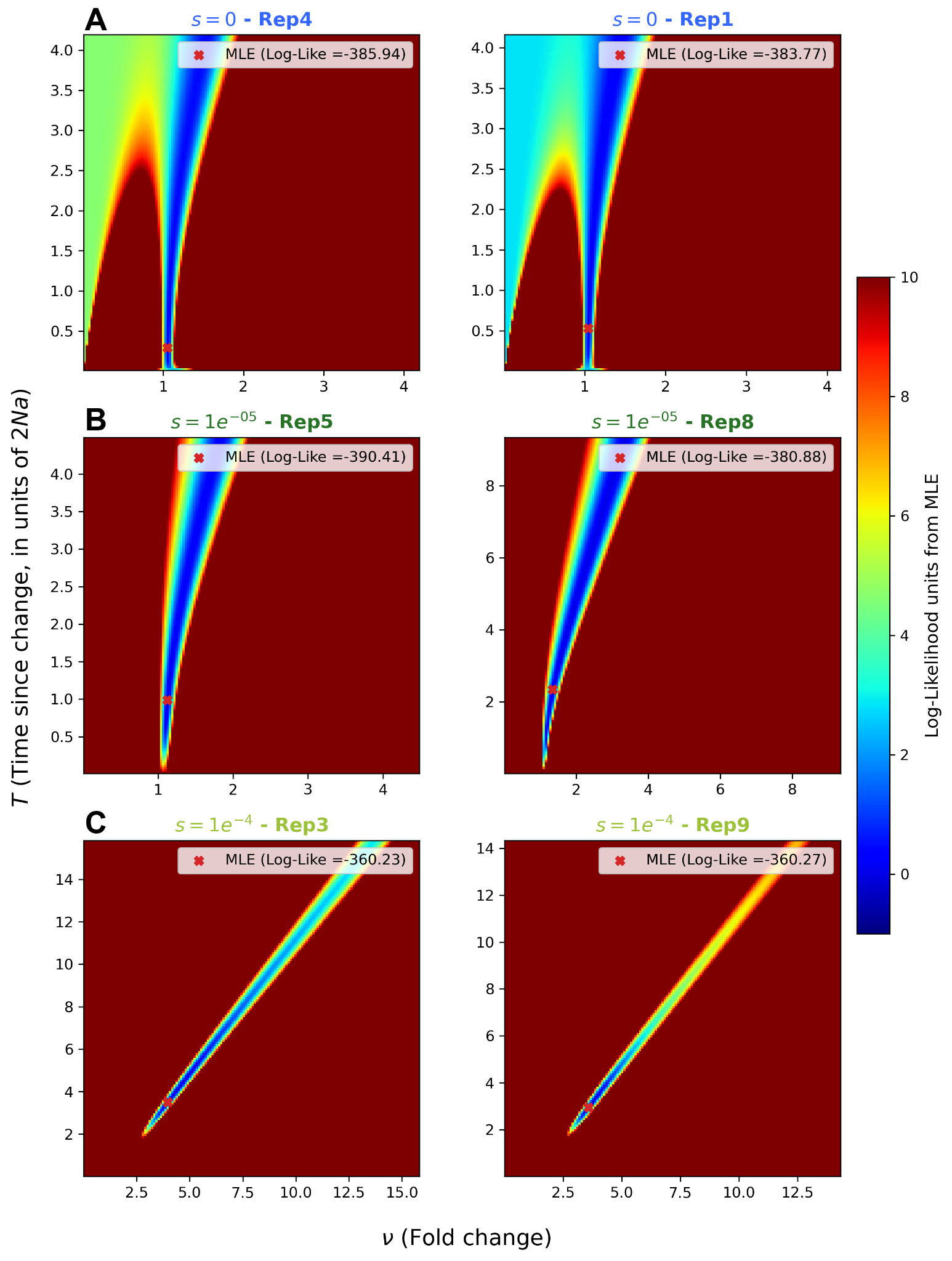


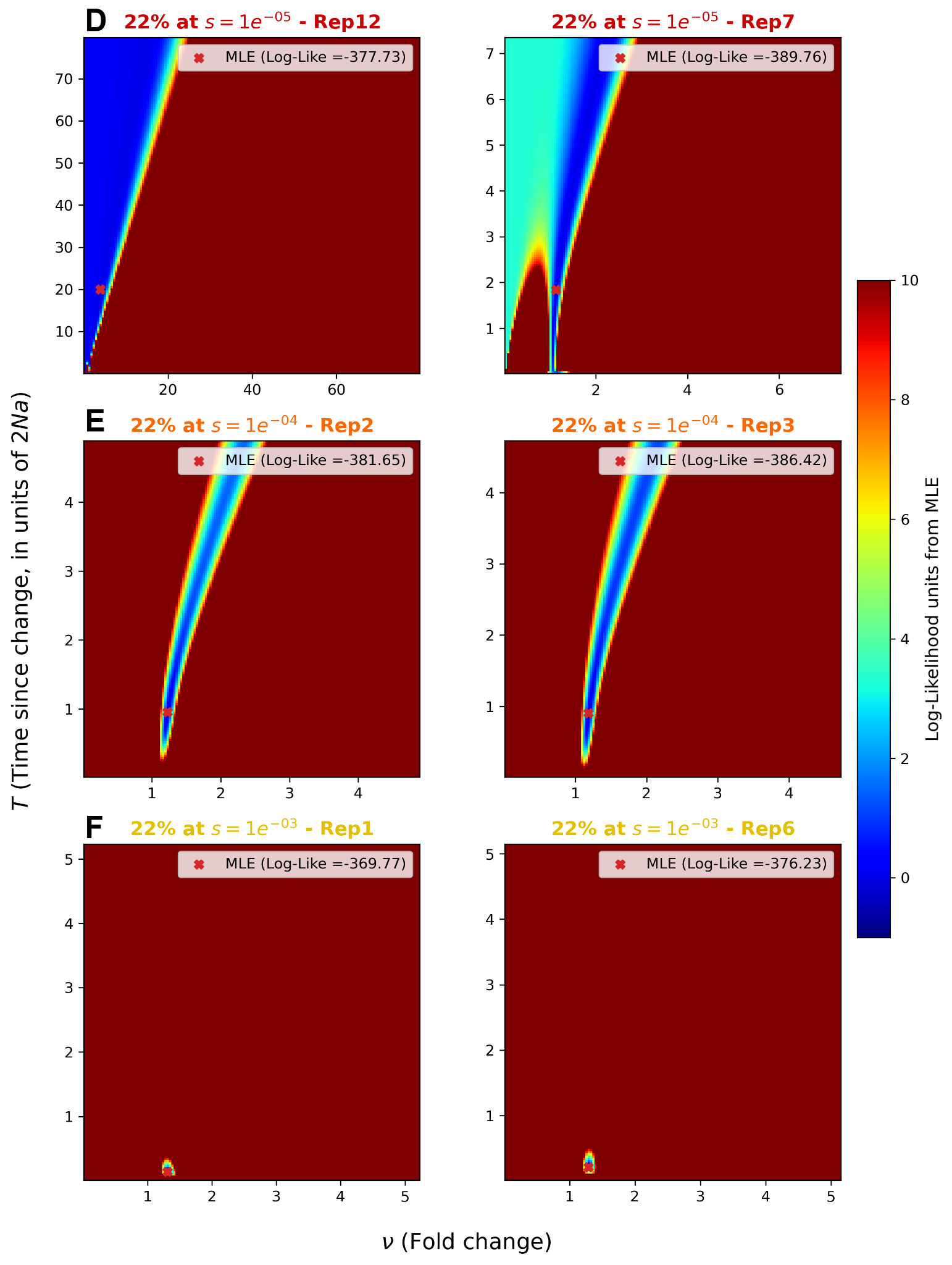


**Supplementary Figure 3: Log-likelihood surfaces for the two-epoch demographic model parameters for data simulated assuming a constant population size**. Each plot shows the log-likelihood surface for a single simulation replicate. The y-axis shows, *T*, the time of the size change in units of *2N_a_* generations. The x-axis represents *v*, the fold difference between current and ancestral population size. Each row, **A-F**, shows two representative replicates for each simulated condition. The title of each plot indicates the strength of selection on synonymous mutations for that given replicate. Log-likelihoods are shown in units from the log-likelihood of the MLE of the demographic parameters for the given replicate. The MLE demographic parameters are indicated with a red cross. Plots of the log-likelihood surfaces for all other simulation replicates are available in the GitHub repository associated with this work.
