# Supplementary material for: The impact of non-neutral synonymous mutations when inferring selection on nonsynonymous mutations
Source: Genetics. 2025 Sep 27;231(4):iyaf200. doi: 10.1093/genetics/iyaf200 (PMC12693584; doi:10.1093/genetics/iyaf200)
Supplement: iyaf200_Supplementary_Data [file iyaf200_supplementary_data.zip › Supplementary_Figure_4_GENETICS-2025-308515.docx]

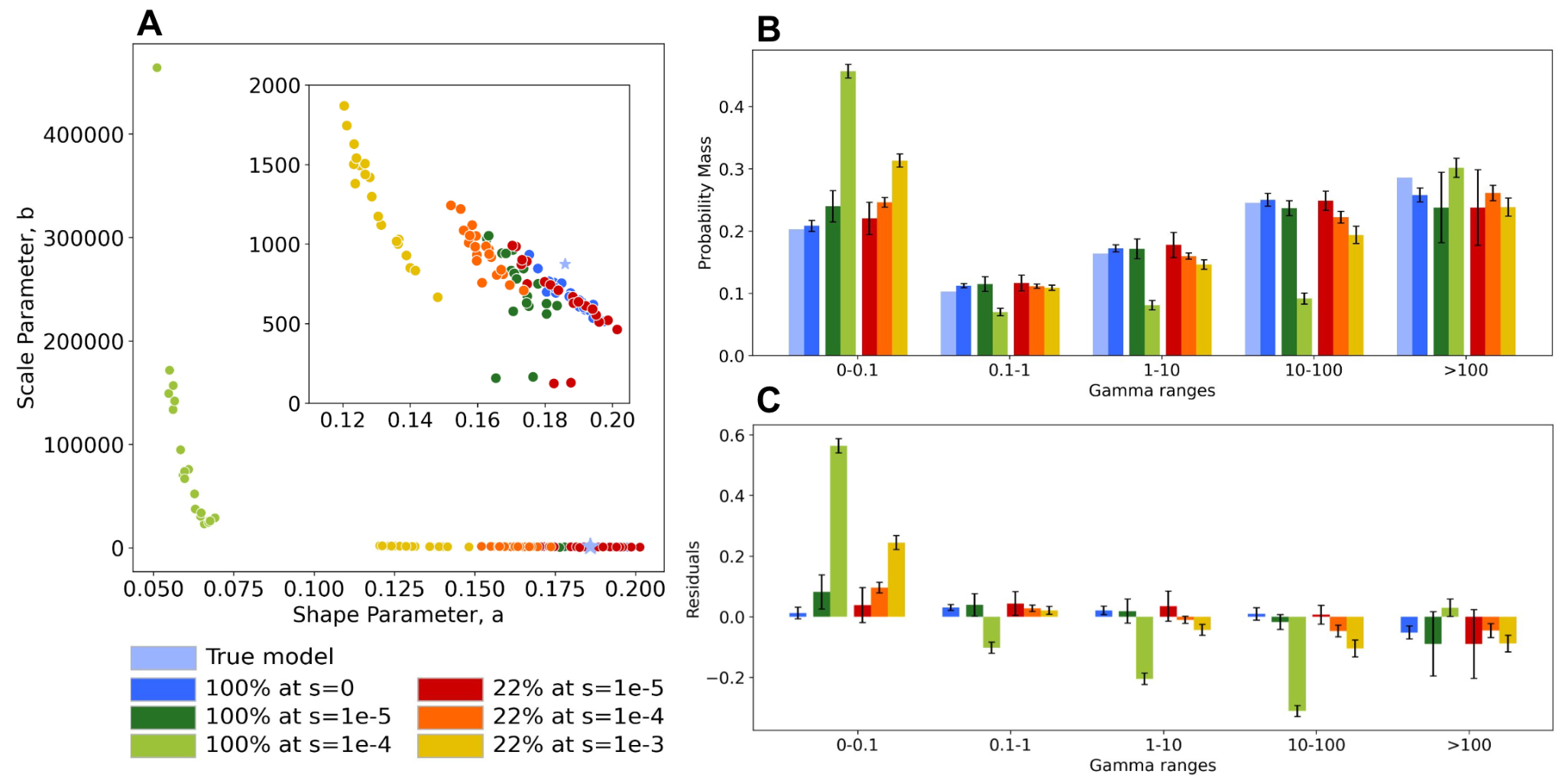


**Supplementary Figure 4: Inference of the DFE for nonsynonymous mutations under different models of selection on synonymous mutations based on the population-scaled selection coefficient (γ).** Results from Figure 2 were obtained by dividing the estimates in terms of γ by the ancestral population size (see Methods). Here we present the results in terms of γ directly. **A** Inferred shape and scale parameters for a gamma DFE model for nonsynonymous mutations from simulated data with distinct levels of selection on synonymous mutations. Each point represents an individual simulation replicate. Scale parameter, *b*, represents the scale parameter in units of population-scaled selection strength. *b* relates to *s_dhet_* through scaling by 2*N_a_*, *s_dhet_* = *b / 2N_a_*. Insert zooms in on the lower-left section of the plot. **B** Comparison of the discretized DFE for nonsynonymous mutations between the true DFE (light blue) and the average inferred DFE for each model of selection on synonymous mutations. Bars represent an average over 20 replicates and error bars show the standard deviation. DFE bins range from neutral (0-1) to strongly deleterious (>100). **C** Standardized residuals of the probability mass in each DFE bin, obtained by subtracting the true probability mass (light blue in B) from the average for each condition, for each bin, divided by the square root of the true probability mass.
