# Supplementary material for: The impact of non-neutral synonymous mutations when inferring selection on nonsynonymous mutations
Source: Genetics. 2025 Sep 27;231(4):iyaf200. doi: 10.1093/genetics/iyaf200 (PMC12693584; doi:10.1093/genetics/iyaf200)
Supplement: iyaf200_Supplementary_Data [file iyaf200_supplementary_data.zip › Supplementary_Figure_5_GENETICS-2025-308515.docx]

**
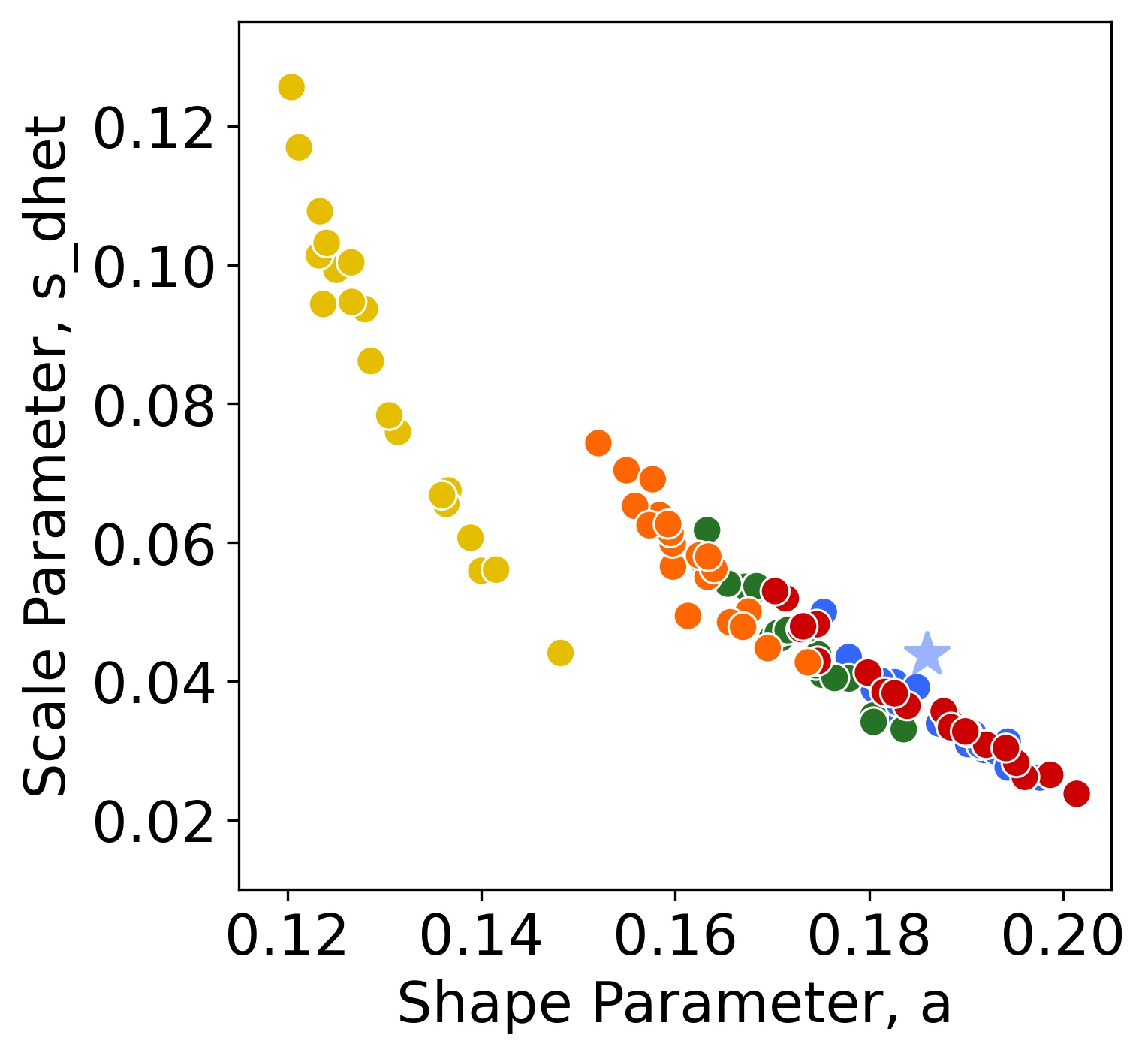
**

**Supplementary Figure 5: Inferred shape and scale parameters in a gamma DFE model for non-synonymous mutations from simulated data with distinct levels of selection on synonymous mutations, zooming in around the true parameter values**. Each point represents an individual simulation replicate. Scale parameter, *s_dhet_*, represents the scale parameter in units of heterozygous selection strength. This figure is the same as Figure 2, but zooms into the area around the true DFE parameters (blue star).
