# Supplementary material for: The impact of non-neutral synonymous mutations when inferring selection on nonsynonymous mutations
Source: Genetics. 2025 Sep 27;231(4):iyaf200. doi: 10.1093/genetics/iyaf200 (PMC12693584; doi:10.1093/genetics/iyaf200)
Supplement: iyaf200_Supplementary_Data [file iyaf200_supplementary_data.zip › Supplementary_Figure_6_GENETICS-2025-308515.docx]

**
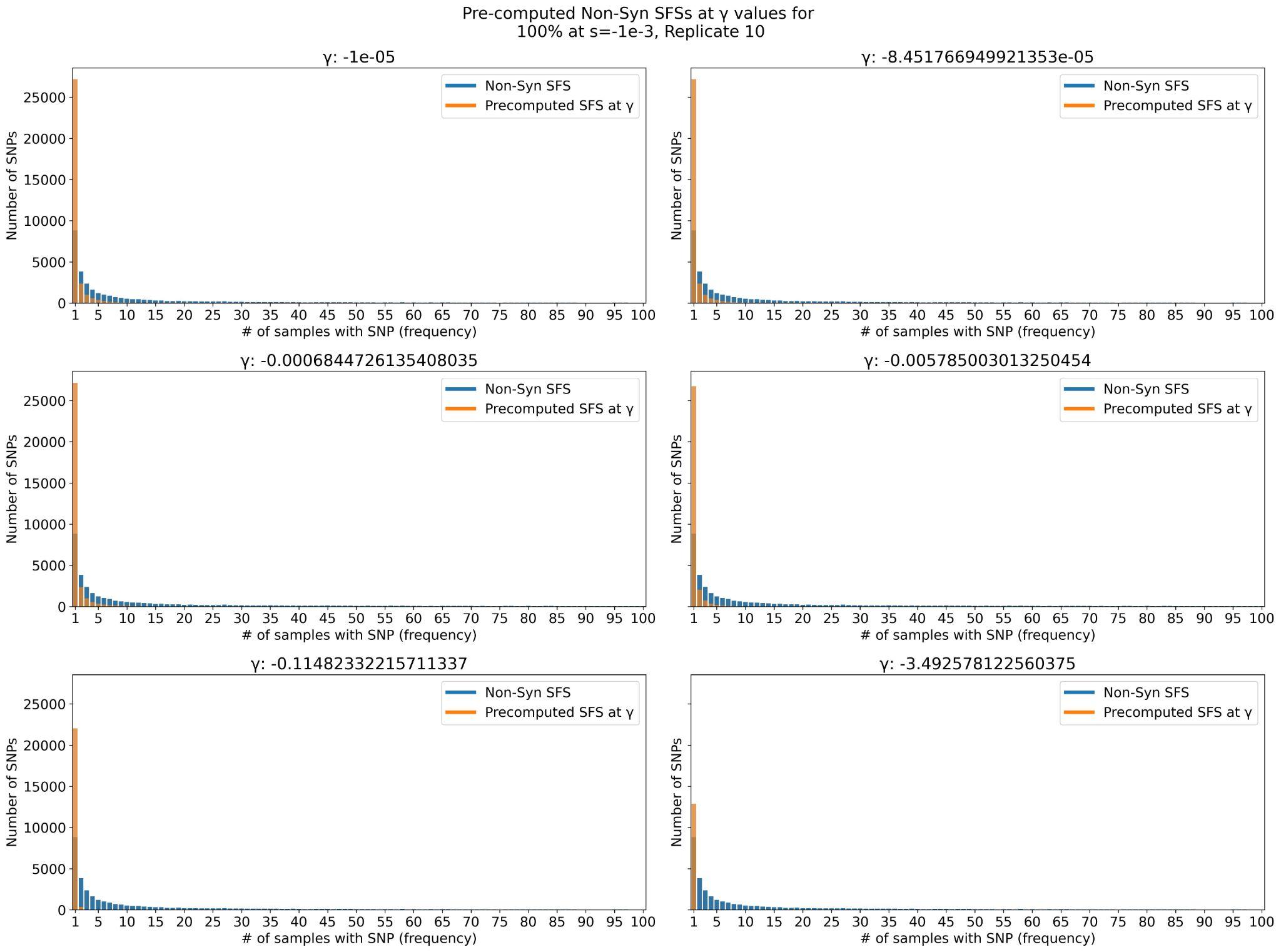
**

**Supplementary Figure 6: Pre-computed SFSs under γ values along the entire γ range for a replicate under the most extreme case of selection on synonymous sites, where 100% of mutations have *s*=1e-3**. Fit∂a∂i pre-computes the expected non-synonymous SFS under γ values ranging from 1e-05 to 2**N_a_**0.5, where 0.5 is the most extreme selection coefficient possible since γ is the population-scaled selection coefficient of the heterozygote. The computation takes into account the demographic model, which was inferred from synonymous variants. None of the pre-computed non-synonymous SFSs (orange) include any common variants (frequency >15) that are seen in the real non-synonymous SFS (blue). This suggests that the inferred demographic model from synonymous variants is so biased that no values of γ for nonsynonymous mutations yield a reasonable fit to the SFS.
