# Supplementary material for: The impact of non-neutral synonymous mutations when inferring selection on nonsynonymous mutations
Source: Genetics. 2025 Sep 27;231(4):iyaf200. doi: 10.1093/genetics/iyaf200 (PMC12693584; doi:10.1093/genetics/iyaf200)
Supplement: iyaf200_Supplementary_Data [file iyaf200_supplementary_data.zip › Supplementary_Figure_7_GENETICS-2025-308515.docx]

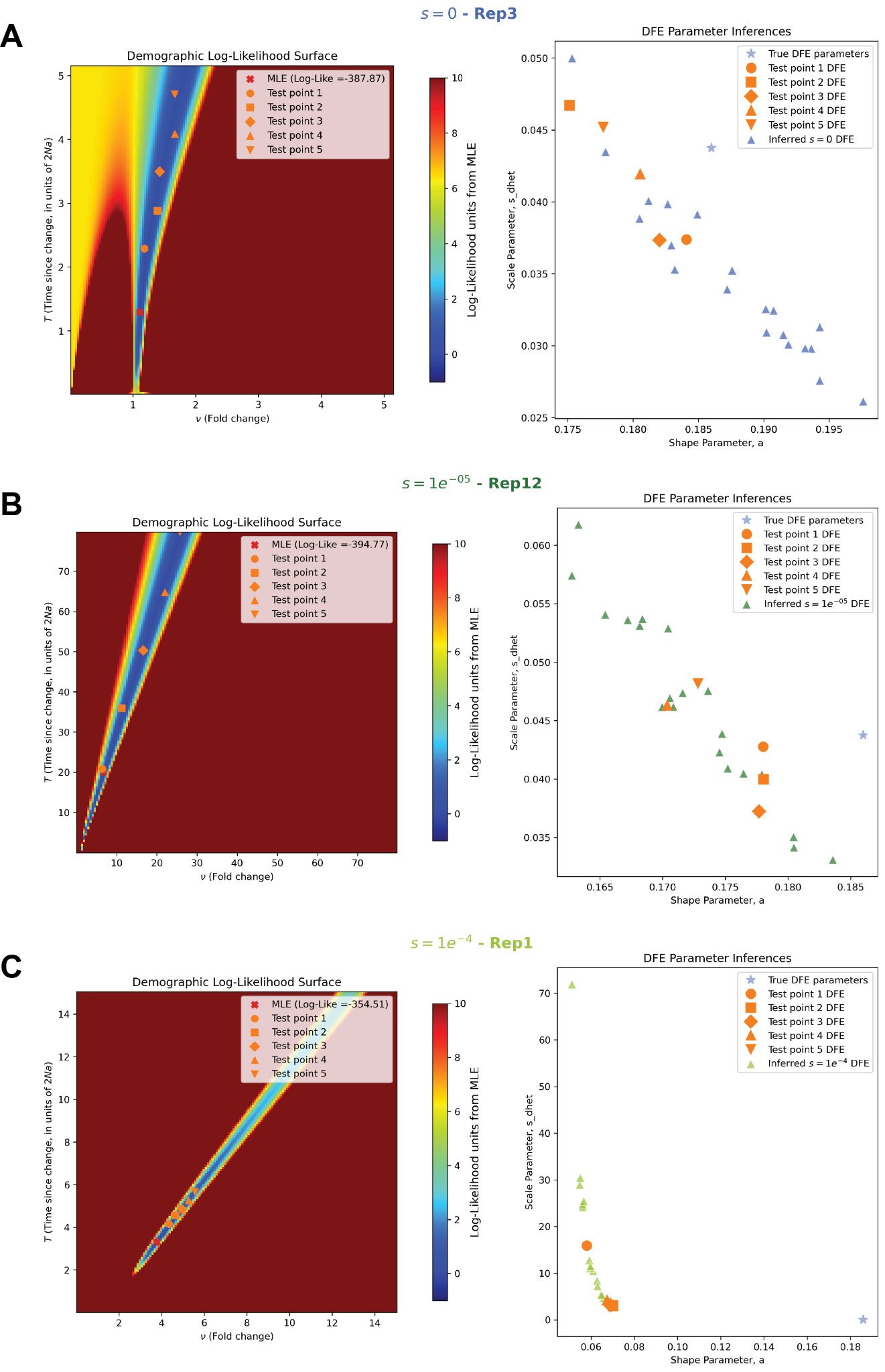


**
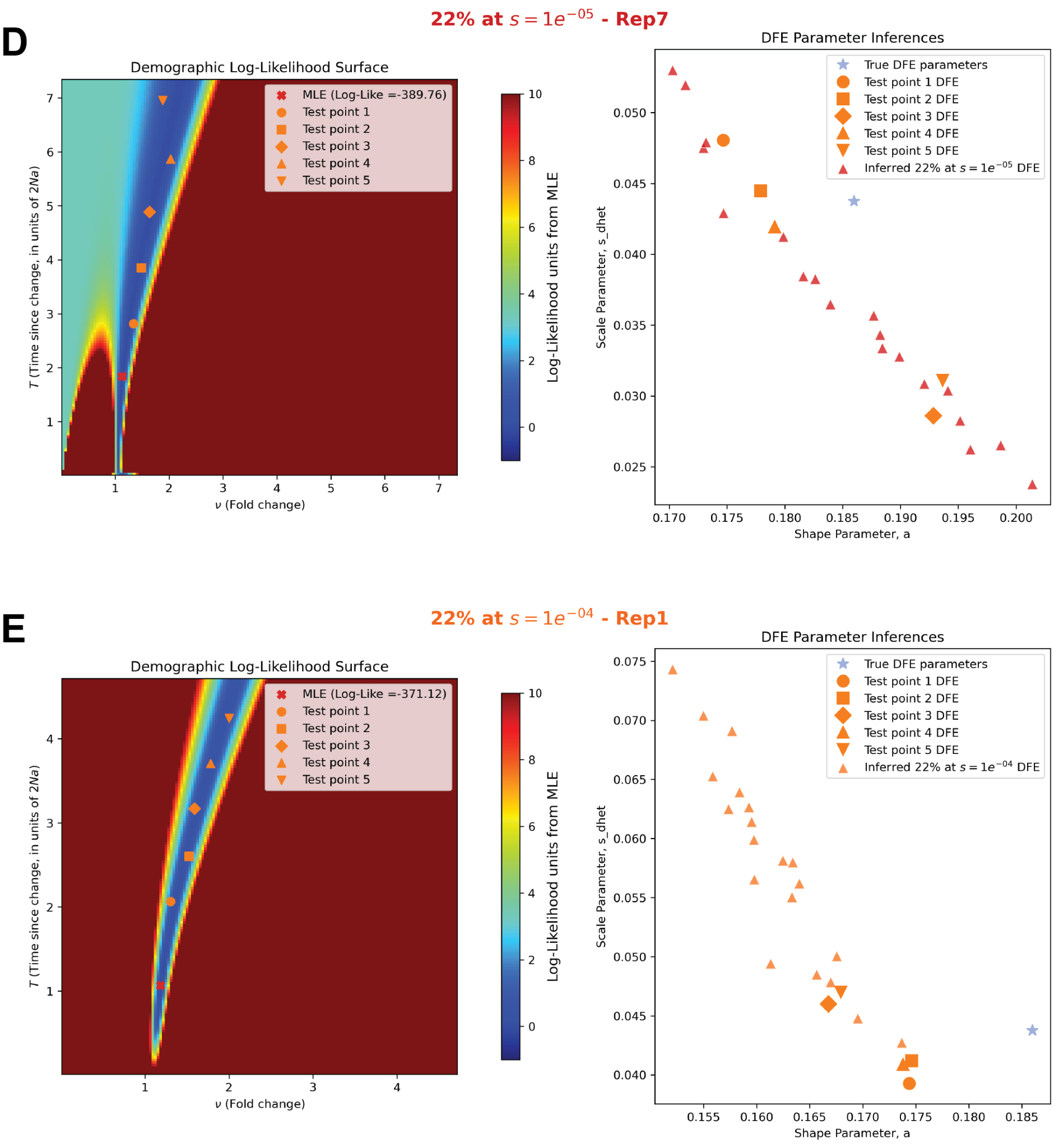
**

**Supplementary Figure 7: Inferred nonsynonymous DFE parameters for a range of demographic models.** Each pair of plots, **A-E**, represents the results for a single replicate with a constant population size. The title of each plot indicates the strength of selection on synonymous mutations for that given replicate. Within a replicate, the left graph shows the log-likelihood surface of the two-epoch demographic model parameters. The maximum likelihood estimate (MLE) parameters are shown with a red cross. 5 other possible demographic parameter combinations that fell within 1 log-likelihood unit of the log-likelihood at the MLE are shown in orange. These parameter combinations were then used for DFE inference. The right panels show the inferred shape and scale parameters of the gamma DFE model for nonsynonymous mutations. The inferred DFE parameters obtained when conditioning on a given set of demographic parameters in the left plot are represented by a matched shape orange marker. The true DFE used in the simulations is indicated by a light blue star. DFE parameters obtained across all simulation replicates for the simulation condition of the replicate are shown with upright triangles. Note there is less variability of the DFE parameter estimates when assuming different demographic models compared to the DFE parameter estimates from different simulation replicates, suggesting that demographic models that fit the data yield robust estimates of the DFE parameters.
