# Supplementary material for: The impact of non-neutral synonymous mutations when inferring selection on nonsynonymous mutations
Source: Genetics. 2025 Sep 27;231(4):iyaf200. doi: 10.1093/genetics/iyaf200 (PMC12693584; doi:10.1093/genetics/iyaf200)
Supplement: iyaf200_Supplementary_Data [file iyaf200_supplementary_data.zip › Supplementary_Figure_8_GENETICS-2025-308515.docx]

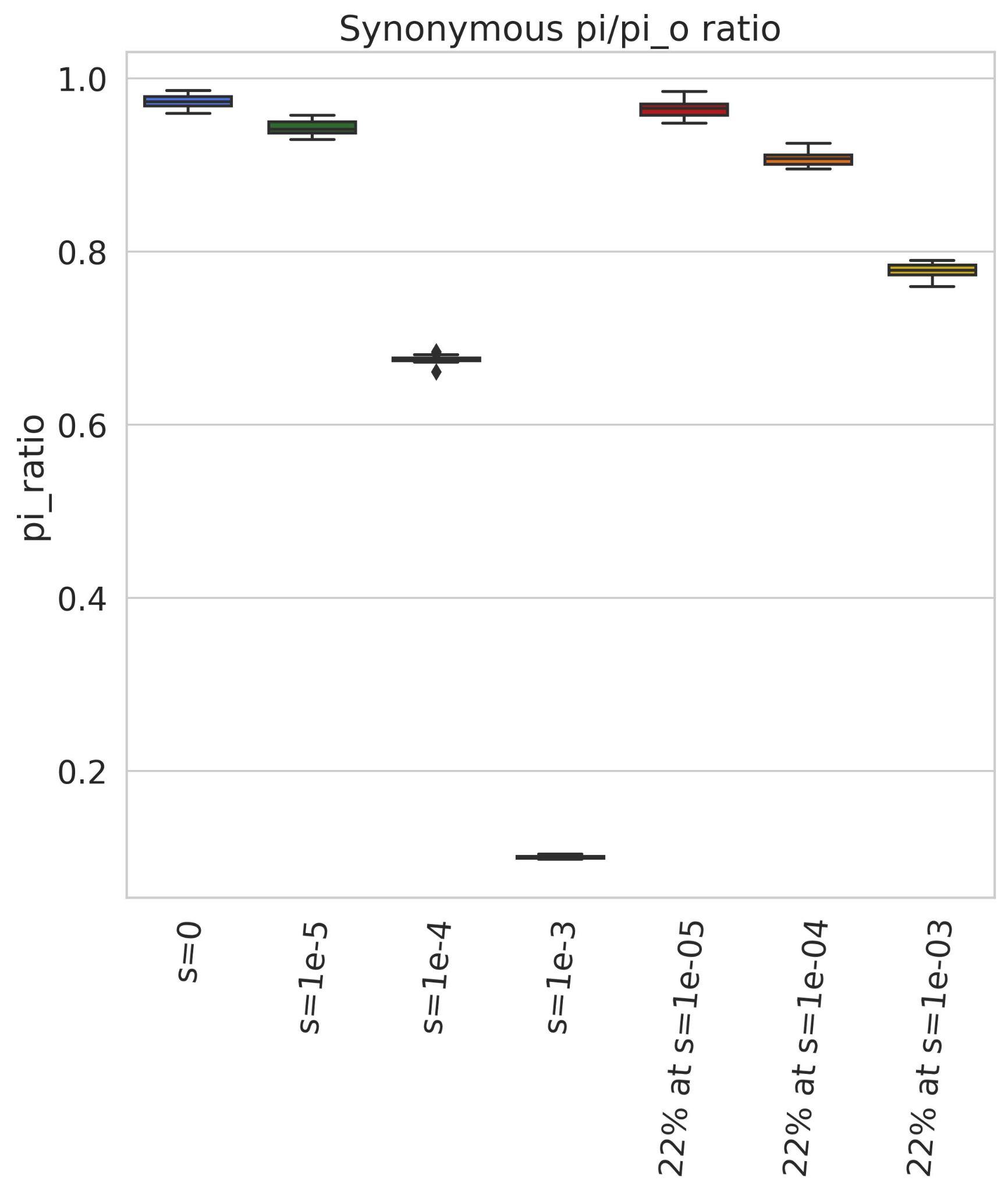


**Supplementary Figure 8: Ratio of observed to expected number of synonymous pairwise differences between two chromosomes (π / π_0_).** For each simulation replicate, π is computed from the SFS and π_0_ is equal to 4N_a_L_ex_𝜇_s_ (See Methods). For each condition, the box indicates the IQR of the distribution, with the horizontal line inside the box indicating the median value. Whiskers extend to the farthest point within 1.5 units of the IQR. Outliers are plotted. Each condition contains 20 replicates.
