# Supplementary material for: The impact of non-neutral synonymous mutations when inferring selection on nonsynonymous mutations
Source: Genetics. 2025 Sep 27;231(4):iyaf200. doi: 10.1093/genetics/iyaf200 (PMC12693584; doi:10.1093/genetics/iyaf200)
Supplement: iyaf200_Supplementary_Data [file iyaf200_supplementary_data.zip › Supplementary_Figure_9_GENETICS-2025-308515.docx]

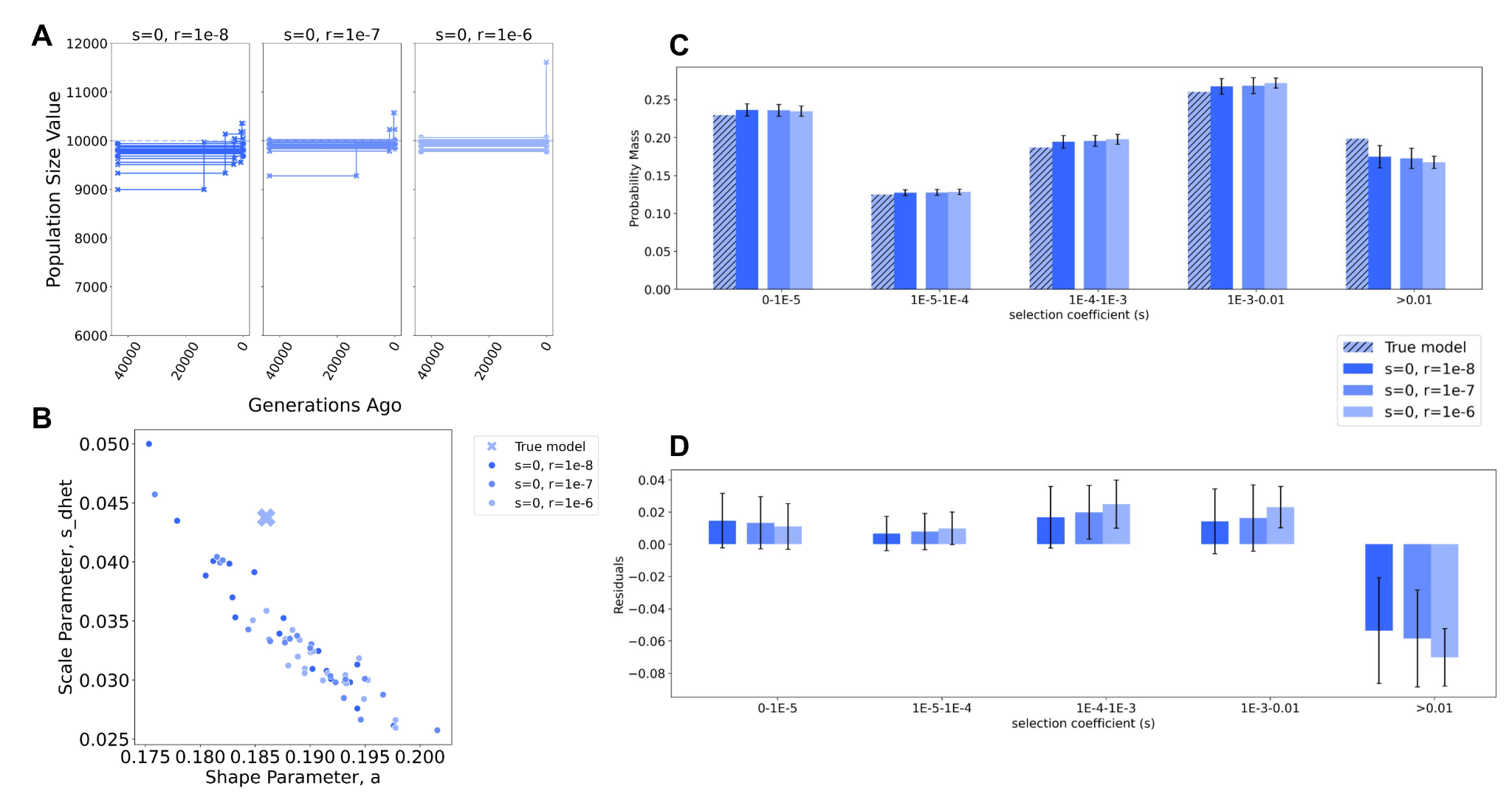


**Supplementary Figure 9:** **Inference of demographic and DFE parameters when synonymous mutations are neutral under varying rates of recombination (*r*)**. All replicates simulated with *s*=0 on synonymous mutations. Different shades of blue represent different rates of recombination. **A** Inferred population size for each replicate under each recombination rate tested. **B** Inferred shape and scale parameters for a gamma DFE model for nonsynonymous mutations from simulated data with distinct rates of recombination. Each point represents an individual simulation replicate. Scale parameter, *s_dhet_*, represents the scale parameter in units of heterozygous selection strength. **C** Comparison of the discretized DFE for nonsynonymous mutations between the true DFE (striped blue) and the average inferred DFE. Bars represent an average over 20 replicates, error bars show standard deviations. DFE bins range from neutral (0-1E-5) and nearly neutral (1E-5-1E-4) to strongly deleterious (>0.01). **D** Standardized residuals of the probability mass in each DFE bin, obtained by subtracting the true probability mass (striped blue in C) from the average for each condition, for each bin, divided by the square root of the true probability mass.
