# Supplementary material for: The impact of non-neutral synonymous mutations when inferring selection on nonsynonymous mutations
Source: Genetics. 2025 Sep 27;231(4):iyaf200. doi: 10.1093/genetics/iyaf200 (PMC12693584; doi:10.1093/genetics/iyaf200)
Supplement: iyaf200_Supplementary_Data [file iyaf200_supplementary_data.zip › Supplementary_Table_1_GENETICS-2025-308515.docx]

***Supplementary Table 1: Simulation parameters.***

Population genetic parameters used for forward-in-time simulations. The parameters are as follows: *n_c_*, number of haploid chromosomes sampled to obtain the SFS of a particular simulation run; *N_a_*, ancestral population size in diploids; 𝜇, mutation rate per base pair per generation; *r*, recombination rate per base position per generation; *L_t_* total genomic length simulated per simulation run; *L_e_*, average exonic length simulated per simulation run, with standard error. The length is reported as an average due to the exon/intron pairs being randomly generated for each simulation run. *L_ns_/L_s_* is the ratio of possible nonsynonymous to synonymous mutations, obtained from Huber et al. 2017. *L_er_* is the average exonic length per replicate, after aggregating 22 simulations.

| *n_c_* | *N_a_* | *𝜇* | *r* | *L_t_* | *L_e_* | *L_ns_/L_s_* | *L_er_* |
| --- | --- | --- | --- | --- | --- | --- | --- |
| 100 | 10000 | 1.5e-8 | 1e-8 | 1e8 | 1463488 +/- 1585 | 2.31 | 32206526  +/- 7102 |
