# Supplementary material for: The impact of non-neutral synonymous mutations when inferring selection on nonsynonymous mutations
Source: Genetics. 2025 Sep 27;231(4):iyaf200. doi: 10.1093/genetics/iyaf200 (PMC12693584; doi:10.1093/genetics/iyaf200)
Supplement: iyaf200_Supplementary_Data [file iyaf200_supplementary_data.zip › Supplementary_Table_3_GENETICS-2025-308515.docx]

***Supplementary Table 3: Comparison of the one and two epoch model fits across simulation replicates.*** The table lists the number of replicates (out of a total of 20) for which a one epoch or two epoch model provided the best fit to the data. The first column indicates the model of selection on synonymous sites used in the simulations. The second column indicates the recombination rate used in the simulations. For the remainder of the replicates simulated with selection acting on synonymous mutations, which are not listed here, a two-epoch model always provided the best fit to the data.

| Model of selection on synonymous sites | Recombination rate | One epoch demographic model is best | Two epoch demographic model is best |
| --- | --- | --- | --- |
| 100% with *s*=0 | 1e-8 | 14 | 6 |
| 100% with *s*=0 | 1e-7 | 16 | 4 |
| 100% with *s*=0 | 1e-6 | 19 | 1 |
| 22% with *s*=1e-5 | 1e-8 | 9 | 11 |
| 22% with *s*=1e-5 | 1e-7 | 10 | 10 |
| 22% with *s*=1e-5 | 1e-6 | 14 | 6 |
